# The effectiveness of interventions on clinical and patient-reported outcomes in hospital-to-home transitions of older adults: a systematic review

**DOI:** 10.1007/s10433-025-00890-w

**Published:** 2025-11-04

**Authors:** Laura Maria Steiner, Selvedina Osmancevic, Sabine Hahn, Loris Bonetti, Sandra Zwakhalen

**Affiliations:** 1 Department of Business Economics, Health and Social Care, University of Applied Sciences and Arts of Southern Switzerland, Manno, Switzerland; 2 Nursing Research Competence Centre, Department of Nursing, Ente Ospedaliero Cantonale, Bellinzona, Switzerland; 3 Institute of Nursing Science, Medical University of Graz, Graz, Austria; 4 Applied Research and Development in Nursing, Department of Health Professions, Bern University of Applied Sciences, Bern, Switzerland; 5 Department of Health Services Research, Care and Public Health Research Institute CAPHRI, Faculty of Health, Medicine and Life Sciences, Maastricht University, Maastricht, Netherlands

**Keywords:** Hospital readmission, Older adults, Patient discharge, Transitional care, Systematic review

## Abstract

The global ageing population presents growing challenges for health and social care systems, particularly during transitions from hospital to home for older adults. These high-risk periods are associated with functional decline, hospital readmissions, and reduced quality of life. This systematic review synthesised evidence from 25 randomised controlled trials (RCTs), involving 17,542 participants aged 65 and older, to evaluate the effectiveness of transitional care interventions. A comprehensive search of PubMed, CINAHL Complete, and Scopus was conducted for studies published between January 2013 and March 2024. Outcomes were categorised using the Core Outcome Measures in Effectiveness Trials (COMET) taxonomy into four domains: clinical outcomes, life impact, resource use, and death/adverse events. Most interventions were multicomponent, combining structured discharge planning, caregiver involvement, follow-up, and home-based support, typically delivered by multidisciplinary teams. Interventions were frequently associated with improvements in functional status, mood, and nutritional outcomes, as well as modest reductions in hospital readmissions and emergency department visits in the short term. Few studies assessed outcomes beyond six months, and sustained effects were uncommon. Few interventions demonstrated effects on mortality or adverse events. Caregiver engagement, particularly involving informal caregivers, and early post-discharge follow-up emerged as key enablers of success. Due to considerable heterogeneity in intervention design and outcomes, findings were synthesised narratively. This review highlights the potential of personalised, interdisciplinary transitional care models tailored to the complex needs of older adults. Future research should prioritise long-term outcomes, patient-reported experiences, and the development of scalable, context-sensitive strategies, including digital tools and caregiver-inclusive approaches.

**Supplementary Information:**

The online version contains supplementary material available at 10.1007/s10433-025-00890-w.

## Introduction

The ageing global population presents increasing challenges for health and social care systems, particularly in facilitating safe hospital-to-home transitions. By 2050, an estimated 22% of the global population will be aged 65 or older, with even higher proportions expected in Europe (Eurostat 2025; WHO 2024). As people age, they are more likely to experience multiple chronic conditions, and complex care needs often result in repeated transitions between healthcare settings (Laugaland et al. 2012; Leithaus et al. 2022). These transitions, particularly from hospital to home, are high-risk periods associated with adverse outcomes such as medication errors, unplanned readmissions, and functional decline (Leithaus et al. 2022; Leppin et al. 2014). Such complications can seriously compromise older adults’ health autonomy and ability to age in place (Liebzeit et al. 2021). Limited health literacy may further hinder patients’ understanding of discharge instructions and medication regimens, contributing to poor adherence and increased risk of readmission (Boyle et al. 2017).

Transitional care refers to a set of actions designed to ensure the safe, coordinated, and continuous transition of healthcare as patients move between different locations or levels of care (Coleman et al. 2006). Effective transitional care is particularly important for older adults returning home after acute hospitalisation, a period marked by vulnerability and uncertainty. Hospital-to-home transitions are clinically vulnerable periods, associated with risks such as medication errors, functional decline, and readmissions. They are also deeply personal experiences, as older adults adjust to changes in independence, routines, and available support at home. Transitional theory highlights how personal readiness, available resources, and social support shape older adults’ experiences during these transitions (Meleis 2010). This theoretical lens is particularly relevant to our review, as it underlines why both clinical outcomes (e.g., Quality of life (QoL), autonomy, and self-management) are essential to capture the full impact of transitional care. These elements are also central to concepts such as ageing in place and successful ageing, which depend on maintaining independence, autonomy, and well-being in the home setting (WHO 2015). A wide range of interventions has been developed to support these transitions. Common strategies include structured discharge planning, medication reconciliation, patient and caregiver education, follow-up telephone calls, and home visits. Typically delivered by nurses, pharmacists, or interdisciplinary teams, these interventions aim not only to reduce immediate risks but also to promote self-management and enable older adults to live independently for as long as possible (Gough et al. 2022; Hestevik et al. 2019; Hudson et al. 2014; Meleis 2010). When implemented effectively, transitional care interventions can reduce complications, enhance patient safety, and improve satisfaction and continuity of care, while also reducing healthcare costs (Morkisch et al. 2020; Pauly et al. 2018).

While previous systematic reviews have yielded important insights, they have predominantly focused on narrowly defined populations (e.g., patients with heart failure, dementia, or stroke) or have examined a limited set of outcomes, typically readmissions and mortality (Braet et al. 2016; Gonçalves-Bradley et al. 2022; Jee et al. 2022; Lee et al. 2023; Parker et al. 2020; O’Callaghan et al. 2022; Li et al. 2021). For example, a recent Cochrane review found that structured discharge planning probably leads to modest reductions in length of stay and readmissions, but its effects on mortality, patient-reported health outcomes, and costs remain limited or uncertain (Gonçalves-Bradley et al. 2022). However, broader health-related outcomes, such as QoL, functional status, self-management, and caregiver burden, have received comparatively less attention, despite being critical to the independence and well-being of older adults (Hirschman et al. 2015; Marengoni et al. 2011; Muth et al. 2019). This lack of focus on patient-centred outcomes has been highlighted in a scoping review by Liebzeit et al. (2021), which found that most transitional care interventions continue to prioritise system-level metrics, such as readmission rates and length of stay. This points to a clear gap in the literature. A more comprehensive synthesis is needed, one that includes both clinical and patient-reported outcomes. Unlike previous reviews limited to diagnoses such as heart failure or stroke, this review considers a broader group of older adults aged 65 and over returning home after acute hospitalisation.

To address this gap, this systematic review synthesises existing evidence on the effectiveness of transitional care interventions for older adults aged 65 and over, specifically those returning home after an acute hospital stay. Unlike previous reviews that focused on specific diagnoses or a narrow set of outcomes, this review includes a broader range of populations and evaluates health-related outcomes, including both clinical (e.g., readmissions, length of stay, and mortality) and patient-reported outcomes (e.g., QoL, satisfaction, self-management capacity, and caregiver burden). The research question for this review is: What is the effectiveness of interventions designed to optimise the transition of older adults from an acute care setting to home on a comprehensive range of health-related outcomes, including clinical outcomes (e.g., readmissions, mortality, and length of stay) and patient-reported outcomes (e.g., QoL, satisfaction, self-management, and caregiver burden)?

## Methods

This systematic review followed the Preferred Reporting Items for Systematic Review and Meta-analyses (PRISMA) guidelines (Page et al. 2021). The review protocol was registered in PROSPERO (registration ID: CRD42024494576).

### Eligibility criteria

Studies were included if they met the following criteria:

- *Population:* Older adults were defined as individuals aged 65 years or older, consistent with public health and policy definitions in Europe and internationally (WHO 2015).
- *Setting*: Transitions from acute care, defined as short-term, intensive medical care typically provided in hospitals for urgent or severe conditions, are recognised as high-risk periods for older adults.
- *Intervention:* Transitional care interventions delivered at different stages of the hospital-to-home transition: pre-discharge interventions, delivered before the patient leaves hospital; bridging interventions (during hospitalisation) that link hospital and community care services, and post-discharge interventions initiated after the patient returns home.
- *Comparator:* Usual or standard care, defined as routine discharge and follow-up normally provided within the healthcare system, without additional structured transitional support.
- *Outcomes:* Health-related outcomes, defined as both clinical measures (e.g., hospital readmissions, length of stay, and mortality) and patient-reported outcomes (e.g., functional ability, symptom management, and QoL). We also considered healthcare utilisation and caregiver burden as relevant outcomes, although these are not patient-reported measures.
- *Study design:* Randomised controlled trials (RCTs). Although our protocol allowed both RCTs and quasi-experimental designs, we ultimately included only RCTs. Full-text screening identified 25 eligible RCTs, which provided a strong evidence base to address our review objectives. RCTs are the “gold standard” for evaluating intervention effectiveness because they minimise selection bias, confounding, and other threats to internal validity. Quasi-experimental designs, by relying on non-random allocation, make causal attribution more difficult and introduce heterogeneity in quality, design, and risk of bias, reducing comparability across studies. As the Cochrane Handbook (Chapter 24) advises, non -randomised studies should be included only when RCT evidence is lacking. Given the robust number of RCTs, we therefore restricted our review to RCTs (Reeves et al. 2023).
- *Language:* Publications in English, German, French, or Italian.

Studies were excluded if they:

- Focused on patient groups with defined care pathways, such as those living in institutional settings (e.g., nursing homes or long-term care facilities), patients receiving palliative or end-of-life care, or those with primary mental health diagnoses (e.g., dementia).
- Used ineligible designs, including non-randomised trials, observational studies, and descriptive research, as these designs lack comparable internal validity.
- Reported only non-patient-related health outcomes, such as staff compliance or documentation rates.
- Were published in languages other than English, German, French, or Italian, due to translation limitations.

### Search strategy

A comprehensive literature search was conducted across three databases: PubMed, CINAHL, and Scopus. We searched for studies published between January 2013 and March 2024 to capture recent evidence reflecting current transitional care practices and policy in transitional care. The search strategy combined keywords and Medical Subject Headings (MeSH) related to older adults, transitional care, hospital discharge, and relevant outcomes. An information specialist (KS) reviewed and refined the search strategy for accuracy, and the final search string was adapted for each database. Only peer-reviewed journal articles were included in the review. The full search strategy is available in Appendix 1 Table 1.

**Table 1:** Critical appraisal of the included experimental studies (JBI Critical appraisal checklist for RCTs)

Reference	Q1	Q2	Q3	Q4	Q5	Q6	Q7	Q8	Q9	Q10	Q11	Q12	Q13	Rating (N of yes out of 13)
Alizadeh-Khoei 2023	Y	Y	Y	Y	N	Y	N	Y	U	Y	U	Y	Y	9
Altfeld et al. 2013	Y	U	Y	U	U	Y	U	Y	U	Y	Y	Y	Y	8
Arendts et al. 2018	Y	N	Y	N	N	N	Y	Y	U	N	Y	Y	Y	7
Berglund et al. 2015	Y	U	Y	U	U	N	U	Y	Y	Y	Y	Y	Y	8
Biese et al. 2014	Y	U	Y	U	N	N	N	U	U	Y	U	Y	Y	5
Biese et al. 2018	Y	Y	Y	N	N	Y	Y	Y	Y	Y	Y	Y	Y	11
Blondal et al. 2023	Y	Y	Y	N	N	Y	Y	Y	Y	Y	U	Y	Y	10
Buurman et al. 2016	Y	Y	Y	Y	U	Y	Y	Y	Y	Y	Y	Y	Y	12
Clemson et al. 2016	Y	Y	Y	N	N	N	Y	Y	Y	Y	Y	Y	Y	10
Deer et al. 2019	U	U	Y	U	U	Y	N	Y	U	N	Y	N	U	4
Deutz et al. 2016	Y	Y	Y	Y	Y	Y	Y	Y	Y	Y	Y	Y	Y	13
Finlayson et al. 2018	Y	Y	Y	N	U	Y	Y	Y	U	Y	Y	Y	Y	10
Grahn et al. 2019	Y	U	Y	Y	N	U	N	Y	U	N	U	Y	Y	6
Gurwitz et al. 2014	Y	Y	Y	U	U	U	Y	Y	Y	Y	U	Y	Y	9
Jepma et al. 2021	Y	Y	Y	Y	N	U	Y	Y	Y	Y	Y	Y	Y	11
Kempen et al. 2021	Y	U	Y	N	N	Y	Y	Y	Y	Y	Y	Y	Y	10
Lee et al. 2023	Y	Y	Y	N	N	Y	Y	Y	U	Y	Y	Y	Y	10
Lembeck et al. 2019	Y	Y	Y	N	Y	Y	Y	U	U	Y	Y	Y	Y	10
Lindegaard Pedersen et al. 2017	Y	Y	Y	N	N	Y	N	U	Y	Y	Y	Y	Y	9
Lockwood et al. 2019	Y	Y	N	N	N	Y	Y	Y	Y	Y	Y	Y	Y	10
Ong et al. 2016.	Y	Y	Y	U	N	Y	U	Y	N	Y	Y	Y	Y	9
Ozaki et al. 2023.	U	U	Y	Y	N	Y	U	Y	U	Y	U	Y	Y	7
Schapira et al. 2022.	Y	Y	Y	N	N	Y	Y	Y	Y	Y	Y	Y	Y	11
Van Spall et al. 2019	Y	N	Y	N	N	Y	Y	Y	U	Y	Y	Y	Y	9
Xueyu et al. 2017	N	U	Y	Y	N	U	N	Y	Y	N	U	Y	Y	6
% Yes	88	60	96	28	8	60	68	88	52	84	72	96	96	

Critical appraisal of the included experimental studies (JBI Critical Appraisal Checklist for Randomised Controlled Trials)

Y=Yes, N=No, U=unclear: JBI Critical appraisal Checklist for Randomised Controlled Trials.: Q1= Was true randomization used for assignment of participants to treatment groups?; Q2: Was allocation to treatment groups concealed?; Q3: Were treatment groups similar at baseline?; Q4= Were participants blind to treatment assignment?; Q5: Were those delivering the treatment blind to treatment assignment?; Q6: Were treatment groups treated identically other than the intervention of interest?; Q7: Were outcome assessors blind to treatment assignment?; Q8: Were outcomes measured in the same way for treatment groups?; Q9: Were outcomes measured in a reliable way?; Q10: Was follow-up complete and if not, were differences between groups in terms of their follow-up adequately described and analysed?; Q11: Were participants analysed in the groups to which they were randomised?; Q12: Was appropriate statistical analysis used?

### Study selection

**Figure 1:**
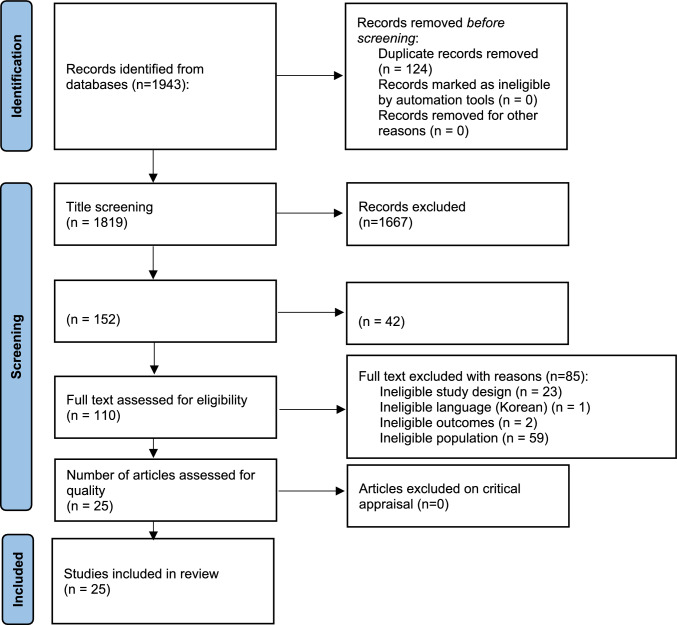
PRISMA flow diagram (Page et al. 2021)

All retrieved citations were imported into Mendeley Reference Manager (Mendeley Ltd., Elsevier, Netherlands) for duplicate removal. Two reviewers (LMS and SO) independently screened titles and abstracts, followed by full-text screening of potentially eligible studies. Screening was managed through the JBI SUMARI platform (Joanna Briggs Institute System for the Unified Management, Assessment, and Review of Information), which supports standardised workflows for systematic reviews (Munn et al. 2019). Discrepancies were resolved through discussion. No third reviewer was required. The full selection process is illustrated in Fig. 1. Reasons for full-text exclusions, such as ineligible population, study design, language, or outcomes, are listed in Appendix 2.

### Assessment of methodological quality

Methodological quality of included studies was assessed using the JBI Critical Appraisal Checklist for Randomised Controlled Trials available through the SUMARI platform (Barker et al. 2023). This tool evaluates key domains of bias, including sequence generation, allocation concealment, blinding of participants and personnel, group comparability at baseline, the validity and reliability of outcome measures, completeness of follow-up, and selective reporting. Each item was scored as “Yes”, “No”, “Unclear”, or “Not Applicable”. Based on these ratings, studies were classified as having low, high, or unclear overall risk of bias. All included studies were retained for synthesis regardless of their risk rating, in order to provide a comprehensive overview of the available evidence and minimise selective reporting (Page et al. 2021).

### Data extraction

The first author extracted data using the standardised JBI Data Extraction Tool within the JBI SUMARI platform (Munn et al. 2019). A second reviewer (SO) cross-checked the entries for accuracy. Extracted data included study setting, participant characteristics, intervention and comparator details, outcomes assessed, and main findings. A summary of these characteristics is presented in Table 2.

**Table 2:** Characteristics of included studies

Authors country	Setting/design	Population/sample size	Intervention	Control group	Outcome measures		Conclusions
					Primary	Secondary	
(Alizadeh-Khoei et al. 2023) Iran	RCT, hospital	*N* = 304, ≥ 65 yrs with chronic disease	4-week nurse-led intervention starting 24-72hrs pre-discharge, home visit + 2-month calls	Usual care: routine discharge, no follow-up	IADL, ADL, cognition, pain, depression, readmission, QoL	Quality of life (EQ-5D, EQ-VAS)	↑ IADL and QoL; no effect on other outcomes.
(Altfeld et al. 2013) USA	RCT, hospital	*N* = 720, ≥ 65 yrs, complex discharge needs	Post-discharge telephone-based social work care + follow-up survey	Usual care: standard discharge, no proactive contact	Follow-up completion, stress	30-day hospital readmission, 30-day mortality.	Better follow-up; no impact on stress, readmission, or mortality.
(Arendts et al. 2018) Australia	RCT, ED	*N* = 164, ≥ 65 yrs at high risk of ED return	Home visit + follow-up support from nursing/allied health	Usual care: routine GP follow-up	ED reattendance (28 days).	hospitalisation, 1-year outcomes	Modest short-term ED ↓; no long-term effects.
(Berglund et al. 2015) Sweden	RCT, ED	*N* = 161, frail older adults ≥ 65	Continuum of care: ED geriatric assessment + home-based planning	Usual care: standard hospital and municipal care	Life satisfaction (LiSat-11), multiple domains over 12 mo	NR	↑ life satisfaction in function, health, finances at 6–12 mo.
(Biese et al. 2014) USA	3-arm RCT, ED	*N* = 120, ≥ 65 yrs needing outpatient follow-up	Nurse call post-discharge vs. placebo calls vs. no call	All: standard ED discharge	Timely follow-up, med compliance	ED return, costs	↑ follow-up; no difference in return visits or meds
(Biese et al. 2018) USA	RCT, ED	*N* = 2,000, ≥ 65 yrs discharged home	Nurse call 1–3 days post-discharge: meds, barriers, follow-up	Usual care: satisfaction survey, standard discharge	ED return, hospitalisation, death (30 days)	Medication acquisition, follow-up visit completion.	No effect on outcomes or follow-up; simple calls not effective
(Blondal et al. 2023) Iceland	RCT, hospital	*N* = 106, > 65 yrs at nutritional risk	6-month nutrition therapy: 5 home visits, 3 calls, meals/supplements	Usual care: written info, Meals on Wheels referral only	Readmissions, LOS (1–18 mo)	ER visits, mortality, long-term care needs**.**	↓ readmissions, shorter LOS; no effect on ER visits, mortality, or care home use.
(Buurman et al. 2016) Netherlands	RCT, Hospital,	*N* = 674, ≥ 65 yrs at risk of functional decline	Systematic CGA + transitional care (handover + home visits at 2 days, 2, 6, 12, 24 weeks)	Usual care: CGA only during hospital stay	ADL (Katz)	Mortality, cognition, readmission, nursing home discharge	Lower 1- and 6-month mortality; no effect on ADL, cognition, or readmission.
(Clemson et al. 2016) Australia	RCT, 5 hospitals	*N* = 400, ≥ 70 yrs, discharged home	Enhanced OT discharge planning with pre/post home visits & follow-up	Usual care; in-hospital OT planning only.	ADL (NEADL), participation (LLDI), readmissions	Unplanned readmissions, ED visits	More OT input but no effect on ADL, participation or readmissions; routine home visits not recommended.
(Deer et al. 2019) USA	Phase I RCT, single academic hospital	*N* = 100, ≥ 65 yrs, acute medical illness, discharged home	Whey protein, in-home rehab, protein + rehab, or testosterone vs. placebo.	Isocaloric placebo	Short Physical Performance Battery (SPPB)	Body composition, ADL, 30-day readmissions	↑ SPPB and trend towards ↓readmissions; no effect on ADL or body composition; larger trials needed.
(Deutz et al. 2016) USA	Multicentre RCT, hospital and post-discharge	*N* = 652, ≥ 65 yrs, malnourished, hospitalised for CHF, AMI, pneumonia or COPD	High-protein oral supplement with HMB, 2 servings/day for 90 days	Isocaloric placebo	Composite of 90-day death or nonelective readmission	30-/60-day death or readmission, mortality, LOS, nutritional status (SGA), weight, ADL	No effect in composite endpoint or readmission; mortality ↓; nutritional status and weight improved; ADL unchanged.
(Finlayson et al. 2018) Australia	RCT, 2 metropolitan hospitals	*N* = 222, ≥ 65 yrs, medical admission, ≥ 1 readmission risk factor	4 arms: usual care; exercise only; nurse home visit + calls (N-HaT); exercise + nurse home visit + calls (ExN-HaT)	Usual care	Unplanned hospital readmission at 28 days, 12 weeks, 24 weeks	Functional ability, psychosocial well-being	Multifaceted interventions (N-HaT, ExN-HaT) ↓ readmissions at 28 days and 12 weeks vs control; effect not sustained at 24 weeks; exercise alone ineffective.
(Grahn et al. 2019) USA	RCT, 3 hospitals, colorectal surgery patients with new ileostomy	*N* = 100, elective or nonelective ileostomy	Enhanced compliance surveillance of an ileostomy education program, with staff prompts and post-discharge monitoring	Usual care, with standard education program only	Unplanned hospital readmission within 30 days	Readmission for dehydration or AKI, any acute renal failure, direct costs, patient satisfaction	↑ follow-up and outpatient IV use but no reduction in readmissions or AKI; intervention was cost neutral and did not affect satisfaction.
(Gurwitz et al. 2014) USA	RCT, large multispecialty group practice	*N* = 3,661 discharges (≥ 65 yrs) to home	EHR-based automated alerts for providers/staff: discharge notice, medication changes, interaction warnings, and prompts for timely follow-up	Usual care without automated alerts	Outpatient primary care visit within 7-, 14-, and 30-days post-discharge	30-day rehospitalisation	No significant effect on follow-up visits rates or readmission; simple EHR alerts alone not sufficient to improve outcomes.
(Jepma et al. 2021) Netherlands	Single-blind RCT, 6 hospitals	*N* = 306, ≥ 70 yrs, high-risk cardiac	Nurse-coordinated transitional care: CGA, handover, nurse visits, home-based CR	Usual cardiac care ± centre-based CR	6-mo unplanned readmission or mortality	Composite at 3 & 12 mo; readmission & mortality separate at 3, 6, 12 mo	No effect on primary outcome; mortality ↑ at 12 mo
(Kempen et al. 2021) Sweden	Cluster RCT, 8 wards in 4 hospitals	*N* = 2,637, ≥ 65 yrs	CMR alone or CMR + post-discharge follow-up (calls, referral)	Usual care (no pharmacist)	Unplanned hospital visits (admissions + ED) at 12 mo	ED visits, med-related admissions, PCC visits, time to event, mortality, costs	No benefit for unplanned visits; CMR+ Follow-up ↑ ED use; alternative CMR models needed
(Lee et al. 2023) South Korea	Single-blind RCT, tertiary hospital	*N* = 32, frail ≥ 65 yrs.	Frailty-focused transitional care (admission care, structured discharge, home visit + 6 calls)	Usual care	Health service use: readmission, unplanned ER visits	Function, symptoms, depression, nutrition, self-rated health, empowerment, connectedness	Effective for some outcomes (↓ unplanned ER, ↓ depression); no effect on readmission; further tailoring needed
(Lembeck et al. 2019) Denmark	Single-centre RCT, rural hospital	*N* = 537, frail ≥ 65 yrs.	Discharge planning + single follow-up home visit (nurse + municipal nurse)	Usual care	Unplanned readmission at 8, 30, 180 days	Time to first readmission, no of readmissions, LOS, ACSC readmission, GP visits, municipal services, mortality	No effect of single home visit; more intensive interventions likely needed for frail elderly
(Lindegaard-Pedersen et al., 2017) Denmark	RCT, home-based follow-up	*N* = 208, ≥ 75 yrs, malnourished or at risk, living alone	3x individualised nutritional follow-ups: home visits or phone calls (dietician + home carer)	Usual care + diet plan, no follow-up	Acute readmission at 30 & 90 days	Mortality (reported), ADL change (powered for but not primary outcome here)	Home visits ↓ readmission; phone may help if fully delivered; personalised nutrition follow-up effective for frail older adults
(Lockwood et al. 2019) Australia	RCT, hospital wards + community	*N* = 77, ≥ 50 yrs, hip fracture	Pre-discharge home assessment visit by OT	Usual care	Falls, readmissions at 30 day & 6 mo	FIM, SMAF, NEADL, EQ-5D, FES-I	Home visit ↓ short-term readmission, ↑ functional independence; may ↓ falls risk; no other differences
(Ong et al. 2016) USA	Multicentre RCT, 6 academic hospitals	*N* = 1,437, ≥ 50 yrs, HF discharge	Predischarge HF education + nurse phone coaching + remote telemonitoring	Usual care	180-day all-cause readmission	30-day readmission, 30- & 180-day mortality, QoL	Remote monitoring and coaching did not ↓ readmission; slight QoL benefit; no mortality effect
(Ozaki et al. 2023) Japan	Cluster RCT, 19 clinics	*N* = 112, frail ≥ 65 yrs with home care → acute hospitalisation	Early care info transfer: standardised referral template at admission	Usual care, Usual referral letter only	Quality of care transition (4 domains)	Patient satisfaction (HPSQ-13)	No effect on early info transfer; no effect on care quality or satisfaction; better tech & processes needed
(Schapira et al. 2022) Argentina	Single-blind RCT, tertiary hospital	*N* = 240, frail ≥ 75 yrs	Geriatric co-management + interdisciplinary transitional care (CGA, tailored plan, home counsellor follow-up)	Usual care; senior internal medicine + standard discharge options.	30-day readmission	6-mo ER visits, 6-mo mortality	Geriatric co-management + transitional care ↓ readmission & ER visits; promising for frail older adults in Latin America
(Van Spall et al. 2019) Canada	Stepped-wedge cluster RCT, 10 hospitals	*N* = 2,494, ≥ 50 yrs, HF discharge	Patient-centred transitional care: nurse-led self-care education, discharge summary, PCC follow-up <1 wk, home visits + HFC for high-risk	Usual care, clinician discretion	Composite: 3-mo readmission, ED visit, or death; 30-d readmission or ED visit	Discharge preparedness (B-PREPARED), transition quality (CTM-3), QoL (EQ-5D-5L), QALY	Did not ↓ readmission, ED use or mortality; ↑ patient-reported preparedness & transition experience
(Xueyu et al. 2017) China	Single-blind RCT, tertiary hospital	*N* = 78, ≥ 70 yrs, stable CHF	12-wk low-intensity walking protocol + standard transitional care	Usual care; no exercise	HRQoL (MLHFQ), 6MWD, TUG	HR, LVEF	Low intensity walking during transition ↑ QoL & physical function; safe & feasible for older CHF patients

*Symbol/Abbr. * ↑, increased/improved; ↓, decreased/reduced; +, and; AMI= Acute Myocardial Infarctation; AKI, Acute Kidney Injury; CHF, Congestive Heart Failure; CMR, Comprehensive Medication Review; GP, General Practitioner; HF= Heart Failure; HFC, Heart Failure Clinic; HMB, β-Hydroxy β-Methylbutyrate; HRQoL, Health-related Quality of Life; LOS= Length of Stay; mo, months; no., Number; NR, Not Reported; OT, Occupational Therapist; PCC, Primary Care Provider; QoL, Quality of Life; wk, Week; yrs, Years

*Care interventions and Programmes*: CTI, Care Transition Intervention; CGA, Geriatric Comprehensive Assessment; PACT-HF, Patient-Centred Transitional Care Service for Heart Failure

Cognitive and Psychological Scales: CES-D (IOWA), Centre for Epidemiologic Studies Depression Scale (Iowa); CPS, Cognitive Performance Scale; DRS, Disability Rating Scale; GDS, Geriatric Depression Scale; MMSE, Mini-Mental State Examination; PHQ-9, Patient Health Questionnaire-9

Functional and Daily Living Scales: 6MWD, 6-Minute Walking Distance; ADL, Activities of Daily Living; FIM, Functional Independence Measure; IADL, Instrumental Activities of Daily Living; Katz ADL, Katz Index of Independence in Activities of Daily Living; Lawton IADL, Lawton Instrumental Activities of Daily Living; LLDI, Late Life Disability Index; NEADL, Nottingham Extended Activities of Daily Living; SMAF, Functional Autonomy Measurement System; SPPB, Short Physical Performance Battery; TUG, Timed Up and Go; WI-Q, Walking Impairment Questionnaire

Health, Quality of Life and Satisfaction Surveys: B-PREPARED, Brief Scale Measuring Patient Preparedness for Hospital Discharge; CTM-3, Care Transition Measure, 3-item version; EQ-5D, European Quality of Life Questionnaire 3-level version; EQ-5D-5L, European Quality of Life Questionnaire 5-level version; EQ.VAS, European Quality of Life Questionnaire Visual Analogue Scale; HES, Health Empowerment Scale; HPSQ-13, Handwriting Proficiency Screening Questionnaire; LiSat-11, Life Satisfaction Questionnaire; MLHFQ, Minnesota Living with Heart Failure Questionnaire; NHP, Nottingham Health Profile; RHDS, Readiness for Hospital Discharge Scale; S-CAHPS, Consumer Assessment of Healthcare Providers and Systems Surgical Care Survey; SAS, Symptom Assessment Scale; SF-12, Short Form Health Survey Questionnaire; VAS, Visual Analogue Scale

Malnutrition and Frailty Scales: DEXA, Dual-Energy X-ray Absorptiometry, EFS, Edmonton Frail Scale; FES-I, Falls Efficacy Scale International; MNA, Mini Nutritional Assessment; PG-SGA, Patient-Generated Subjective Global Assessment; SGA, Subjective Global Assessment

Social and Family Support Scales, Family APGAR, Family Adaptability, Partnership, Growth, Affection, and Resolve Test; MOS, Medical Outcomes Study Social Support Survey

### Data synthesis

Due to the substantial heterogeneity in interventions, outcome measures, and follow-up durations, we did not conduct a meta-analysis. Instead, findings were synthesised using a structured narrative approach. To aid interpretation and comparison across studies, outcomes were categorised according to the core outcome measures in effectiveness trials (COMET) taxonomy (Dodd et al. 2023). This taxonomy groups outcomes into five core domains: clinical outcomes (such as readmission or symptom control), life impact (including QoL and caregiver burden), resource use (such as healthcare utilisation or costs), death (including all-cause mortality), and adverse events (such as safety concerns) (Dodd et al. 2023). Applying this framework helped standardise reporting across studies with diverse outcome profiles.

### Assessing certainty of evidence

We initially planned to assess the overall certainty of evidence using the Grading of Recommendations, Assessment, Development, and Evaluation (GRADE) approach (Schünemann et al. 2023). However, considerable clinical and methodological heterogeneity made it impractical to apply formal GRADE ratings consistently. For example, interventions ranged from nutritional supplementation and exercise programmes to structured discharge planning, home visits, and pharmacist-led medication reviews, with durations spanning from a few days to twelve months. Populations also differed, including frail older adults, disease-specific cohorts (e.g., heart failure, colorectal surgery), and heterogeneous community-dwelling groups, with sample sizes from 32 to over 3600. Outcomes and measurement instruments were equally diverse, and results were inconsistent, with some studies reporting benefits (e.g., reduced readmissions, improved nutrition, or function) while others reported no significant effects. Following guidance from the GRADE Public Health Group (Hilton Boon et al. 2021), we therefore adopted a structured narrative assessment approach to describe heterogeneity, study quality, and consistency of findings. This approach is explicitly recommended when standard GRADE ratings may oversimplify complex evidence, and it provides a rigorous but flexible alternative that preserves contextual detail without reducing findings to oversimplified summary scores.

## Results

### Study selection and characteristics

A total of 1943 records were identified through database searches. After removing duplicates, 1819 titles and abstracts were screened. Of these, 25 RCTs met the inclusion criteria and were included in the final synthesis (Fig. 1). These studies, published between 2013 and 2023, involved 17,542 older adults transitioning from acute care to home across diverse healthcare systems and settings, including medical and surgical hospital wards, emergency departments, and specialised units such as cardiology, geriatrics, and rehabilitation. The study population included both general hospital populations and targeted groups, such as frail, malnourished, chronically ill, and surgical patients. Sample sizes varied substantially, from 32 participants (Lee et al. 2023) to over 3600 (Gurwitz et al. 2014). Most studies compared transitional care interventions with usual care, although two employed placebo-controlled designs (Deer et al. 2019; Deutz et al. 2016). The interventions were typically multicomponent, combining discharge planning, cross-setting coordination, and post-discharge follow-up. Delivery was commonly provided by multidisciplinary teams, including nurses, physicians, physiotherapists, dieticians, occupational therapists, and other allied health professionals. Intervention durations ranged from one week to 12 months, with 21 RCTs falling within the one- to three-month period. Details are provided in Table 2 and Appendix 4 in the Supplement.

### Methodological quality

All studies were appraised using the JBI SUMARI Critical Appraisal Checklist for RCTs (Munn et al. 2019). Studies were rated according to the number of “Yes” responses out of 13 checklist items (high quality: 10–13; moderate: 7–9; low: ≤ 6). Eleven studies were rated as high quality (Biese et al. 2018; Blondal et al. 2023; Buurman et al. 2016; Clemson et al. 2016; Finlayson et al. 2018; Jepma et al. 2021; Kempen et al. 2021; Lee et al. 2023; Lembeck et al. 2019; Lockwood et al. 2019; Schapira et al. 2022), while most others were of moderate quality. The most common limitation was the lack of blinding for participants and treatment providers, an inherent challenge in transitional care trials due to the visible nature of interventions (Altfeld et al. 2013; Arendts et al. 2018; Berglund et al. 2015; Biese et al. 2014; Deer et al. 2019; Grahn et al. 2019; Kempen et al. 2021; Ozaki et al. 2023; Van Spall et al. 2019; Xueyu et al. 2017). Inter-rater agreement between reviewers was 93.85%, with a Cohen’s Kappa value (*k*) of 0.80, indicating near-perfect consistency (Landis and Koch 1977).

### Control group intervention

Across the included studies, usual or standard care typically comprised standard discharge procedures and follow-up, without additional structured support such as nutritional counselling, exercise, home visits, or telemonitoring. However, descriptions of usual care were often limited or lacking detail, making comparisons with intervention arms occasionally difficult to interpret. See Table 2 for further details.

### Intervention characteristics

The content and delivery of transitional care interventions varied but generally followed a three-phase structure: pre-discharge, during hospitalisation, and post-discharge. During hospitalisation, most interventions included comprehensive assessments (e.g., geriatric or nutritional), medication reviews, risk screening, and patient or caregiver education focused on self-care and discharge preparedness. All studies included some form of structured discharge planning. Post-discharge components were common and typically involved home visits, medication management, safety assessments, and telephone follow-up to monitor patient progress and reinforce care plans. Some interventions also incorporated physical exercise, nutritional supplementation, or emotional and cultural support. See Appendix 3 for further details on intervention components and transition stages. Only one study (Ozaki et al. 2023) did not include a post-discharge component. Most interventions were delivered by interdisciplinary teams, with nurses most frequently acting as coordinators. The professional lead varied by intervention type, with other leads including physiotherapists (Xueyu et al. 2017), nutritionists (Blondal et al. 2023; Deutz et al. 2016), occupational therapists (Clemson et al. 2016; Lockwood et al. 2019), pharmacists (Kempen et al. 2021), and physicians (Gurwitz et al. 2014). Intervention duration ranged from short-term programmes of 1 or 2 weeks to longer-term follow-up extending up to 12 months, with only one study reporting outcomes at 1 year (Berglund et al. 2015). See Appendix 4 for further details on duration, frequency, and mode of delivery of interventions.

### Main results for primary and secondary outcomes

Outcomes were categorised using the COMET taxonomy into four domains: clinical outcomes, life impact, resource use, death, and adverse events. Complete outcome details and statistical significance are presented in Table 3.

**Table 3:** Intervention measures and outcomes according to the COMET Taxonomy (Dodd et al. 2023)

Outcomes	Quantification (n/N/%)	References	Unit of measure	Results
*Clinical outcomes*				
Cardiac	1/25 (4%)	Xueyu et al. 2017	RHR, LVEF (%)	Not significant^NS^
Metabolism and nutrition nutritional status	3/25 (12%)	Blondal et al. 2023 Deutz et al. 2016 Lee et al. 2023	BMI SGA MNA	Significantly improved^S^ Significantly improved^S^ Not significant^S^
Change in body weight	2/25 (8%)	Deer et al. 2019 Deutz et al. 2016 Blondal et al. 2023	BMI, Muscle mass BMI BMI	Not significant^S^ Significantly increased^S^ Significantly increased^AM^
Energy intake/ protein consumption	1/25 (4%)	Blondal et al. 2023	Daily food record	Significantly increased^AM^
Cognitive functioning	4/25 (16%)	Buurman et al. 2016 Alizadeh-Khoei et al. 2023 Blondal et al. 2023	MMSE CPS MMSE	Not significant^S^ Significantly improved^NS^ NR^AM^
Symptom experience incl. pain, etc.	2/25 (8%)	Alizadeh-Khoei et al. 2023 Lee et al. 2023	VAS SAS	Not significant^NS^ Not significant^NS^
*Life impact outcomes*				
Activities of daily living Functional status/ independence	9/25 (33%)	Buurman et al. 2016 Clemson et al. 2016 Alizadeh-Khoei et al. 2023 Lockwood et al. 2019 Deer et al. 2019 Lockwood et al. 2019 Arendts et al. 2018 Finlayson et al. 2018 Lee et al. 2023	Katz ADL, ADL NEADL, LLDI ADL, IADL FIM ADL, IADL NEADL, SMAF Lawton IADL, ADL IADL, WI-Q ADL	Not significant^P^ Not significant^P^ Significantly improved^P^ Significantly improved^P^ Not significant^S^ Not significant^S^ NR^NS^ NR^NS^ NR^S^
Physical status	4/25 (16%)	Deer et al. 2019 Xueyu et al. 2017 Blondal et al. 2023	SPPB, DEXA 6MWD, TUG SPPB	Significantly improved^P^ Significantly improved^P^ Significantly improved^AM^
Falls	1/25 (4%)	Lockwood et al. 2019	FES-I	Not significant^P^
Psychosocial status Mood/ Depression	4/25 (16%)	Alizadeh-Khoei et al. 2023 Lee et al. 2023 Blondal et al. 2023 Finlayson et al. 2018	DRS GDS-SF CES-D (IOWA) GDS	Significantly reduced^P^ Significantly reduced^P^ Significantly reduced^AM^ NR^S^
Social support	1/25 (4%)	Finlayson et al. 2018	MOS	NR^S^
Family interaction	1/25 (4%)	Lee et al. 2023	Family APGAR	Significantly improved^S^
Connectedness	1/25 (4%)	Lee et al. 2023	KIS	Significantly improved^S^
Quality of life HRQL	6/25 (24%)	Xueyu et al. 2017 Arendts et al. 2018 Ong et al. 2016 Finlayson et al. 2018 Lockwood et al. 2019 Alizadeh-Khoei et al. 2023	MLHFQ NHP MLHFQ SF-12 EQ-5D; EQ.VAS EQ-5D; EQ.VAS	Significantly improved^P^ Not significant^S^ Significantly improved^S^ NR^S^ NR^S^ Not significant^NS^
Overall life satisfaction	1/25 (4%)	Berglund et al. 2015	LiSat-11	Significantly improved^P^
Self-rated health	2/25 (8%)	Altfeld et al. 2013 Lee et al. 2023	Self-made Q. Self-made Q.	Not significant^P^
Compliance (delivery of care), empowerment	1/25 (4%)	Lee et al. 2023	HES	Significantly improved^S^
Satisfaction Patient satisfaction	1/25 (4%)	Ozaki et al. 2023 Grahn et al. 2019	HPSQ-13 S-CAHPS	Not significant^P^ Not significant^S^
Quality of care transition	2/25 (8%)	Ozaki et al. 2023 Van Spall et al. 2019	Self-made questionnaire CTM-3	Not significant^P^ Significantly improved^S^
*Resource use outcomes*				
Hospital readmission	21/25 (84%)	Alizadeh-Khoei et al. 2023 Grahn et al. 2019 Gurwitz et al. 2014 Kempen et al. 2021 Lee et al. 2023 Lembeck et al. 2019 Ong et al. 2016 Van Spall et al. 2019 Lindegaard et al. 2017 Lockwood et al. 2019 Schapira et al. 2022 Deutz et al. 2016 Finlayson et al. 2018 Blondal et al. 2023 Altfeld et al. 2013 Arendts et al. 2018 Biese et al. 2014 Buurman et al. 2016 Clemson et al. 2016 Deer et al. 2019 Jepma et al. 2021	Number/percentage days	Not significant^P^ Not significant^P^ Not significant^P^ Not significant^P^ Not significant^P^ Not significant^P^ Not significant^P^ Not significant^P^ Significantly reduced^P^ Significantly reduced^P^ Significantly reduced^P^ Significantly reduced^P^ Significantly reduced^P^ Significantly reduced^P^ Not significant^S^ Not significant^S^ Not significant^S^ Not significant^S^ Not significant^S^ Not significant^S^ Not significant^S^
ED visits	7/25 (28%)	Arendts et al. 2018 Blondal et al. 2023 Van Spall et al. 2019 Kempen et al. 2021 Lee et al. 2023 Schapira et al. 2022 Biese et al. 2014	Number/percentage days	Not significant^P^ Not significant^P^ Not significant^P^ Not significant^P^ Significantly reduced^P^ Significantly reduced^P^ Not significant^S^
Length of stay (LOS)	3/25 (12%)	Blondal et al. 2023 Deutz et al. 2016 Lembeck et al. 2019	days	Significantly reduced^P^ Significantly reduced^P^ Not significant^S^
GP follow-up	5/25 (20%)	Altfeld et al. 2013 Biese et al. 2014 Biese et al. 2018 Lembeck et al. 2019 Kempen et al. 2021	number/ percentage	Significantly increased^P^ Significantly increased^P^ Not significant^S^ Not significant^NS^ NR^NS^
Institutionalisation/ need for long-term care	2/25 (8%)	Blondal et al. 2023 Arendts et al. 2018	NHPAA number/ percentage	Not significant^S^ NR^S^
Community resource utilisation	1/25 (4%)	Lee et al. 2023	Self-made Q.	Significantly increased^S^
Readiness for hospital discharge	2/25 (8%)	Lee et al. 2023 Van Spall et al. 2019	RHDS B-PREPARED	NR^S^ Significantly increased^S^
Timeliness of follow-up appointments	1/25 (4%)	Gurwitz et al. 2014	days	Not significant^S^
*Death and adverse events*				
Mortality	13/25 (52%)	Biese et al. 2018 Ong et al. 2016 Van Spall et al. 2019 Buurman et al. 2016 Deutz et al. 2016 Altfeld et al. 2013 Arendts et al. 2018 Blondal et al. 2023 Jepma et al. 2021 Schapira et al. 2022 Lembeck et al. 2019 Kempen et al. 2021 Lockwood et al. 2019	Number/percentage	Not significant^P^ Not significant^P^ Not significant^P^ Significantly reduced^S^ Significantly reduced^S^ Not significant^S^ Not significant^S^ Not significant^S^ Not significant^S^ Not significant^S^ Not significant^NS^ NR^S^ NR^S^
Adverse events (intervention oriented)	1/25 (4%)	Deer et al. 2019 Blondal et al. 2023	Number NCP	Not significant ^S^ NR^AM^

^P^ = primary outcome, ^S^= secondary outcome, ^NS^= not specified, ^NR^, no results, ^AM^=additional measurements

*Outcome measures acronyms: *NHPAA, Nursing Home Pre-Admission Assessment; CES-D (IOWA), Centre for Epidemiologic Studies Depression Scale (Iowa); CPS, Cognitive Performance Scale; DRS, Disability Rating Scale; GDS, Geriatric Depression Scale; MMSE, Mini-Mental State Examination; PHQ-9, Patient Health Questionnaire-9

Functional and Daily Living Scales: 6MWD, 6-Minute Walking Distance; ADL, Activities of Daily Living; FIM, Functional Independence Measure; IADL, Instrumental Activities of Daily Living; Barthel ADL, Activities of Daily Living Scale; Katz ADL, Katz Index of Independence in Activities of Daily Living; Lawton IADL, Lawton Instrumental Activities of Daily Living; LLDI, Late Life Disability Index; NEADL, Nottingham Extended Activities of Daily Living; SMAF, Functional Autonomy Measurement System; SPPB, Short Physical Performance Battery; TUG, Timed Up and Go; WI-Q, Walking Impairment Questionnaire; B-PREPARED, Brief Scale Measuring Patient Preparedness for Hospital Discharge; CTM-3, Care Transition Measure, 3-item version; EQ-5D, European Quality of Life Questionnaire 3-level version; EQ-5D-5L, European Quality of Life Questionnaire 5-level version; EQ.VAS, European Quality of Life Questionnaire Visual Analogue Scale; HES, Health Empowerment Scale; HPSQ-13, Handwriting Proficiency Screening Questionnaire; LiSat-11, Life Satisfaction Questionnaire; MLHFQ, Minnesota Living with Heart Failure Questionnaire; NHP, Nottingham Health Profile; RHDS, Readiness for Hospital Discharge Scale; S-CAHPS, Consumer Assessment of Healthcare Providers and Systems Surgical Care Survey; SAS, Symptom Assessment Scale; SF-12, Short Form Health Survey Questionnaire; VAS, Visual Analogue Scale; DEXA, Dual-Energy X-ray Absorptiometry, EFS, Edmonton Frail Scale; FES-I, Falls Efficacy Scale International; MNA, Mini Nutritional Assessment; PG-SGA, Patient-Generated Subjective Global Assessment; SGA, Subjective Global Assessment

Social and Family Support Scales: Family APGAR, Family Adaptability, Partnership, Growth, Affection, and Resolve Test; MOS, Medical Outcomes Study Social Support Survey

#### Clinical outcomes

Improvements in nutritional status, body weight, and dietary intake were consistently reported in studies that included nutrition-focused interventions (Blondal et al. 2023; Deutz et al. 2016; Deer et al. 2019). Cognitive function was assessed in four studies, with one reporting significant improvement (Alizadeh-Khoei et al. 2023). Outcomes related to symptom experience, including pain (Alizadeh-Khoei et al. 2023; Lee et al. 2023) and cardiac function (Xueyu et al. 2017), were infrequently reported and showed no significant effects.

#### Life impact

Outcomes related to physical performance, mood, and ADLs were commonly assessed, particularly in interventions incorporating exercise or psychosocial support. Several studies reported significant improvements (Deer et al. 2019; Lockwood et al. 2019; Blondal et al. 2023). Outcomes related to health-related QoL improved in a minority of studies, especially those involving home-based components (Xueyu et al. 2017; Ong et al. 2016). Measures such as fall risk, social support, and discharge readiness were less frequently evaluated and yielded mixed findings (Lockwood et al. 2019; Van Spall et al. 2019).

#### Resource use

Hospital readmissions were reported in 21 studies. Six showed statistically significant reductions, often in interventions with early follow-up and structured discharge planning (Lindegaard-Pedersen et al. 2017; Blondal et al. 2023; Deutz et al. 2016; Lockwood et al. 2019; Schapira et al. 2022; Finlayson et al. 2018). Fewer studies showed reductions in ED visits and length of stay (Lee et al. 2023; Schapira et al. 2022; Blondal et al. 2023; Deutz et al. 2016). Some interventions with post-discharge telephone contact were associated with increased GP engagement (Altfeld et al. 2013; Biese et al. 2014). Institutionalisation and community resource use were seldom reported and did not demonstrate consistent effects (Arendts et al. 2018; Blondal et al. 2023; Lee et al. 2023).

#### Mortality and adverse events

Mortality was reported in 13 studies, with significant reductions observed in two (Deutz et al. 2016; Buurman et al. 2016). Adverse events were rarely reported, and no study found significant differences between intervention and control groups (Deer et al. 2019; Blondal et al. 2023).

## Discussion

### Summary of main findings

This systematic review synthesised evidence from 25 RCTs evaluating interventions aimed at optimising the transition from acute care to home for older adults. Overall, multicomponent interventions, particularly those including early follow-up, family involvement, and multidisciplinary delivery, were associated with modest reductions in hospital readmissions, along with improvements in functional status, mood, and QoL. However, these benefits were generally short-term. Most studies reported outcomes within 1 to 3 months after discharge, and only a few extended follow-ups beyond 6 months. These findings align with prior research emphasising the value of comprehensive, person-centred transitional care, while also highlighting persistent challenges in achieving long-term impact and adapting interventions across diverse care settings (Morkisch et al. 2020).

### Key components of effective transitional care

The variation in intervention components across studies reflects the diverse ways transitional care is designed and delivered in practice. Despite this heterogeneity, interventions that combined elements across different time points, such as pre-discharge planning, in-hospital coordination, and post-discharge support, tended to be more effective. Early follow-up, typically within 48 h, structured communication with primary care, and home-based services consistently yielded better outcomes, reinforcing the importance of continuity across care settings (Buurman et al. 2016; Finlayson et al. 2018; Lockwood et al. 2019; Morse et al. 2019; Hestevik et al. 2019). Interdisciplinary collaboration also emerged as a key driver of success. Many effective interventions were delivered by multidisciplinary teams comprising nurses, physiotherapists, social workers, and pharmacists. These teams enabled comprehensive assessment and coordinated care, aligning with previous findings that team-based interventions can enhance patient safety and quality of care (Baxter et al. 2020; Parker et al. 2020). However, as Everall et al. (2019) noted, the effectiveness of such interventions also depends on clearly defined roles, adequate training, and effective communication, factors that were not always well described in the studies reviewed. Although short-term improvements in outcomes such as readmissions, functional status, and psychosocial well-being were common, few studies reported sustained effects beyond 6 months (Alizadeh-Khoei et al. 2023; Lee et al. 2023), underscoring the need for continued support over time.

Where do our findings fit in the literature?

Our findings are consistent with the Cochrane review by Gonçalves-Bradley et al. (2022), which reported that transitional care can reduce readmissions. However, our review extends the existing evidence by including a broader range of intervention types and outcomes, such as nutritional support, psychosocial care, and QoL, areas that have often been underrepresented in previous analyses (Braet et al. 2016; Hirschman et al. 2015). For instance, several studies in our review showed that targeted interventions addressing malnutrition or mobility not only improved clinical outcomes but also contributed to emotional well-being and greater perceived autonomy (Blondal et al. 2023; Deer et al. 2019; Lee et al. 2023). These findings reflect a growing emphasis in gerontological research on “successful ageing”, which prioritises the preservation of function, autonomy, and psychosocial health alongside traditional clinical measures (Meleis 2010; Marengoni et al. 2011). Our findings also support arguments by Leithaus et al. (2022) and Everall et al. (2019) that transitional care is most effective when it addresses the complex and interrelated needs of older adults, rather than focusing solely on system-level outcomes. Interventions that addressed individual risk profiles, through medication reviews, discharge coaching, and caregiver involvement, often achieved better outcomes, highlighting the value of individualised care approaches (Clemson et al. 2016; Alizadeh-Khoei et al. 2023).

Family involvement and informal support as a hidden strength behind successful transitions

Several trials highlighted the added value of engaging informal caregivers in supporting medication adherence, identifying early warning signs, and assisting with daily activities. These findings align with previous research demonstrating the protective effects of caregiver involvement in promoting continuity and safety during care transitions (Gaalen et al. 2021; Ergin et al. 2022). Family participation appears especially critical for older adults with cognitive impairment, mobility limitations, or low health literacy, reinforcing the need to embed caregiver support into transitional care planning (Hudson et al. 2014). However, reliance on informal support raises important equity concerns. As noted by Tyler et al. (2023) and Ong et al. (2016), not all older adults have access to supportive networks, particularly those living alone or in socioeconomically disadvantaged circumstances. To reduce the risk of unequal outcomes, transitional care policies should ensure that tailored support (e.g., increased frequency of home visits, linkages to community volunteer programmes) is available when informal caregiving is limited or absent.

### Are we measuring what really matters?

Only a small number of studies in this review showed statistically significant reductions in mortality, and those that did typically involve highly tailored or intensive interventions (Deutz et al. 2016; Buurman et al. 2016). This reflects broader concerns raised in the literature about the suitability of mortality as a primary outcome in transitional care research (Leppin et al. 2014; Tyler et al. 2023). Mortality is influenced by a wide range of factors, such as baseline health status, care continuity, and social conditions, that often fall outside the scope of transitional interventions. Similarly, while unplanned readmissions are commonly used as a measure of success, they may not always be preventable or undesirable, particularly among older adults with complex multimorbidity.

Several studies included in this review, such as those by Lockwood et al. (2019) and Alizadeh-Khoei et al. (2023), assessed more patient-centred outcomes, such as functional ability, mood, and QoL. These outcomes may more accurately reflect transitional care goals, particularly maintaining independence, well-being, and daily functioning in older adults. The findings add to calls for transitional care research to adopt multidimensional outcome frameworks that go beyond clinical endpoints (Muth et al. 2019; Dodd et al. 2023).

Why setting and resources shape what is possible

The context in which interventions are implemented clearly matters. Most studies in this review were conducted in high-resource settings with access to home-care infrastructure, electronic health records, and community-based support services. Only a few studies (e.g., Ozaki et al. 2023) explicitly examined challenges related to low-resource or rural environments. These disparities echo findings from Lewin et al. (2015), who emphasise the importance of adapting interventions to local conditions, including health system capacity, population literacy, and sociocultural factors. Without such contextual tailoring, even well-designed interventions may be less effective or sustainable without contextual adaptation.

### Strengths and limitations

A strength of this review is its use of the COMET taxonomy to systematically categorise outcomes, allowing a more nuanced interpretation across diverse interventions and populations despite heterogeneous study designs (Dodd et al. 2023). Including studies from varied healthcare systems broadens the applicability of findings. However, several limitations should be acknowledged. Restricting the search to the past 10 years may have excluded relevant earlier studies. Although focusing solely on RCTs improves internal validity and strengthens causal inference, it may reduce external validity by excluding quasi-experimental or observational studies that better reflect real-world implementation, particularly in under-resourced or diverse care settings. In addition, substantial heterogeneity in intervention components, outcome measures, and follow-up periods precluded meta-analysis and limits the generalisability of findings.

## Conclusion

This systematic review offers valuable insights into the effectiveness of interventions supporting the hospital-to-home transition of older adults. While individual components, such as discharge planning and follow-up calls, are well established, this review synthesises the outcome of a wider range of interventions applied to a variety of patient populations. Their effectiveness varied considerably. Personalised care, nutritional support, and structured post-discharge follow-up were associated with improvements in functional status and mood, and in some cases, with modest reductions in readmissions. Multidisciplinary teams were central to intervention delivery and aimed to address the medical, functional, and psychosocial needs of older adults. However, few studies extended follow-up beyond 6 months, which limits conclusions about the sustainability of effects. The diversity in intervention design and outcomes underscores the complexity of transitional care and suggests avenues for future implementation and research, though further study is needed to determine the most effective interventions.

### Recommendations for practice and policy

Based on our synthesis, healthcare systems and practitioners should prioritise transitional care interventions that are personalised, multidisciplinary, and well-coordinated. To move from principle to practice, the evidence supports several concrete actions. Structured, multidisciplinary discharge planning should go beyond standard procedures by establishing a core transitional care team, which may include a coordinating nurse, pharmacist, dietitian, and occupational or physical therapist. Risk assessments should be routinely conducted using validated tools identified in the reviewed studies, such as the mini nutritional assessment (MNA) to detect malnutrition, the Timed Up and Go (TUG) test to assess mobility, and screening for readmission risk factors such as a history of previous hospitalisations. In addition, although none of the included RCTs explicitly evaluated interactive teaching techniques, evidence from the broader health literacy literature supports the use of active education methods such as the “teach-back” method, where, for example, patients or caregivers explain the medication plan in their own words to confirm understanding. Hands-on demonstrations should be provided for any new equipment, and education should be tailored to the patient’s specific condition (for example, symptom monitoring and self-care for heart failure or ileostomy management). We therefore recommend the integration of these strategies into transitional care practice.

### Recommendations for research

Future studies should extend follow-up periods beyond six months, as most existing trials only report short-term effects. Longer observation periods are needed to determine whether benefits on functional recovery, QoL, and mortality are sustained over time. Greater attention should also be given to patient-centred outcomes. The routine use of validated patient-reported outcome measures (PROMs) and patient-reported experience measures (PREMs) would ensure that research reflects what matters most to older adults and their caregivers, moving beyond system-level metrics such as readmissions.

Technology-enabled approaches, including telemonitoring, mobile health applications, and artificial intelligence-supported risk stratification, should be evaluated, particularly in rural or resource-limited settings, where maintaining continuity of care is most challenging.

Future research should also address population diversity and contextual factors. Consideration of health system infrastructure, digital access, socioeconomic conditions, and cultural norms is essential for generating findings that are broadly applicable and equitable. Finally, reproducibility and translation into practice require more transparent reporting. The use of structured frameworks such as the template for intervention description and replication (TIDieR) checklist will support clearer description of interventions and facilitate implementation across different settings.

## Supplementary Information

Below is the link to the electronic supplementary material.

Supplementary Material 1

## Data Availability

The data for this study can be made available upon request to the correspondent author.
