# Supplementary material for: The effectiveness of interventions on clinical and patient-reported outcomes in hospital-to-home transitions of older adults: a systematic review
Source: Eur J Ageing. 2025 Nov 4;22(1):58. doi: 10.1007/s10433-025-00890-w (PMC12586821; doi:10.1007/s10433-025-00890-w)
Supplement: Supplementary file 1 — Supplementary Material 1 [file 10433_2025_890_MOESM1_ESM.docx]

**Supplementary materials**

**Appendix 1**: Search strategy for each database

| **Database** | **Search strategy** |
| --- | --- |
| **PubMed** | ((((("Aged"[MeSH Terms] OR "aged, 80 and over"[MeSH Terms] OR "older adults"[Title/Abstract] OR "geriatric population*"[Title/Abstract] OR "old age"[Title/Abstract] OR "senior*"[Title/Abstract] OR "aging population*"[Title/Abstract] OR "senescence"[Title/Abstract] OR "geriatric patient*"[Title/Abstract] OR "elder*"[Title/Abstract])) AND (("hospital to home transition"[MeSH Terms] OR "patient discharge"[MeSH Terms] OR "transitional care"[MeSH Terms] OR "continuity of patient care"[MeSH Terms] OR "patient admission"[MeSH Terms] OR "discharge plan*"[Title/Abstract] OR "integrated care" [Title/Abstract] OR "patient care plan*" [Title/Abstract] OR "patient pathway*" [Title/Abstract] OR "care transition*"[Title/Abstract]))) AND ((intervention*[Title/Abstract] OR strateg* [Title/Abstract] OR "best practice*" [Title/Abstract] OR program* [Title/Abstract]))) AND (("randomized controlled trials as topic"[MeSH Terms] OR "clinical trials as topic"[MeSH Terms] OR "Non-Randomized Controlled Trials as Topic"[MeSH Terms] OR "pre post test*"[Title/Abstract] OR "quasi experimental*"[Title/Abstract]))) NOT ((review* [Title/Abstract] OR meta analysis [Title/Abstract])) Filters: from 2013 – 2024 |
| **CINAHL complete** | "( AB aged: 65+ years OR AB (older adults or elderly or seniors or geriatrics ) OR AB elderly people ) AND ( AB hospital to home transition OR AB ( patient discharge or hospital discharge or discharge planning ) OR AB ( transitional care or transition of care or care transition or discharge planning ) OR AB ( patient admission or hospitalization or hospital admission ) OR AB continuity of patient care ) AND ( AB interventions or strategies or best practices or treatment or therapy or program or management ) AND ( AB ( clinical trials or randomized controlled trials or controlled clinical trials ) OR AB non-randomized controlled trials OR AB quasi-experimental OR AB ( pre-test and post-test ) ) Publication Date: 20130101-20241231 |
| **Scopus** | ( TITLE-ABS-KEY ( "aged" OR "older adult*" OR "geriatric population*" OR senior* OR "aging population*" OR senescence OR elder* ) ) AND ( TITLE-ABS-KEY ( "hospital to home transition" OR "continuity of patient care" OR "patient admission" OR "discharge plan*" OR "care transition*" ) ) AND ( TITLE-ABS ( intervention* ) ) AND ( TITLE-ABS-KEY ( "randomized controlled trial" OR "clinical trial" OR "quasi experimental" OR "pre post" ) ) AND PUBYEAR > 2012 AND PUBYEAR < 2025 |

**Appendix 2:** JBI Sumari extraction form of excluded studies (shortened version)

| **Excluded studies** |
| --- |
| Aboumatar H, Naqibuddin M, Chung S, Chaudhry H, Kim SW, Saunders J, et al. Effect of a Hospital-Initiated Program Combining Transitional Care and Long-term Self-management Support on Outcomes of Patients Hospitalized with Chronic Obstructive Pulmonary Disease: A Randomized Clinical Trial. 2019;322(14):1380.  **Reason for exclusion: Ineligible participant characteristics** |
| Adam L, Moutzouri E, Baumgartner C, Loewe AL, Feller M, M’Rabet-Bensalah K, et al. Rationale and design of OPtimising thERapy to prevent Avoidable hospital admissions in Multimorbid older people (OPERAM): A cluster randomised controlled trial. 2019;9(6). **Reason for exclusion: Ineligible study design** |
| Baghaei R, Parizad N, Sharifi A, Alinejad V. The effect of continuous nursing care program on anxiety level, episodes of chest pain, and readmission rate after myocardial infarction: A randomized controlled trial. 2021;15(1):28. **Reason for exclusion: Ineligible participant characteristics** |
| Bailey JE, Surbhi S, Wan JY, Munshi KD, Waters TM, Binkley BL, et al. Effect of Intensive Interdisciplinary Transitional Care for High-Need, High-Cost Patients on Quality, Outcomes, and Costs: a Quasi-Experimental Study. 2019;34(9):1824. **Reason for exclusion: Ineligible participant characteristics** |
| Balaban RB, Galbraith AA, Burns ME, Vialle-Valentin CE, Larochelle MR, Ross-Degnan D. A Patient Navigator Intervention to Reduce Hospital Readmissions among High-Risk Safety-Net Patients: A Randomized Controlled Trial. 2015;30(7):915. **Reason for exclusion: Ineligible participant characteristics** |
| Bikmoradi A, Masmouei B, Ghomeisi M, Roshanaei G. Impact of Tele-nursing on adherence to treatment plan in discharged patients after coronary artery bypass graft surgery: A quasi-experimental study in Iran. 2016;86:48. **Reason for exclusion: Ineligible participant characteristics** |
| Black JT, Romano PS, Sadeghi B, Auerbach AD, Ganiats TG, Greenfield S, et al. A remote monitoring and telephone nurse coaching intervention to reduce readmissions among patients with heart failure: study protocol for the Better Effectiveness After Transition - Heart Failure (BEAT-HF) randomized controlled trial. 2014;15(1):124. **Reason for exclusion: Ineligible study design** |
| Bouchand F, Leplay C, Guimaraes R, Fontenay S, Fellous L, Dinh A, et al. Impact of a medication reconciliation care bundle at hospital discharge on continuity of care: A randomised controlled trial. 2021;75(8).  **Reason for exclusion: Ineligible participant characteristics** |
| Bowles KH, Hanlon A, Holland D, Potashnik SL, Topaz M. Impact of discharge planning decision support on time to readmission among older adult medical patients. 2014;19(1):38. **Reason for exclusion: Ineligible participant characteristics** |
| Bronstein LR, Gould P, Berkowitz SA, James GD, Marks K. Impact of a Social Work Care Coordination Intervention on Hospital Readmission: A Randomized Controlled Trial. 2015;60(3):255.  **Reason for exclusion: Ineligible participant characteristics** |
| Cardarelli R, Horsley M, Ray L, Maggard N, Schilling J, Weatherford S, et al. Reducing 30-day readmission rates in a high-risk population using a lay-health worker model in Appalachia Kentucky. 2018;33(1):80. **Reason for exclusion: Ineligible participant characteristics** |
| Carter J, Walton A, Donelan K, Thorndike A. Implementing community health worker-patient pairings at the time of hospital discharge: A randomized control trial. 2018;74:37.  **Reason for exclusion: Ineligible participant characteristics** |
| Chan B, Goldman LE, Sarkar U, Schneidermann M, Kessell E, Guzman D, et al. The Effect of a Care Transition Intervention on the Patient Experience of Older Multi-Lingual Adults in the Safety Net: Results of a Randomized Controlled Trial. 2015;30(12):1794. **Reason for exclusion: Ineligible participant characteristics** |
| Clari M, Frigerio S, Ricceri F, Pici A, Alvaro R, Dimonte V. Follow-up telephone calls to patients discharged after undergoing orthopaedic surgery: Double-blind, randomised controlled trial of efficacy. 2015;24(19):2744.  **Reason for exclusion: Ineligible participant characteristics** |
| Conneely M, Leahy A, O’Connor M, Gabr A, Okpaje B, Saleh A, et al. A physiotherapy-led transition to home intervention for older adults following Emergency Department discharge: a pilot feasibility randomised controlled trial. 2023;23(1):8154.  **Reason for exclusion: Ineligible study design** |
| Coskun S, Duygulu S. effects of Nurse Led Transitional Care Model on elderly patients undergoing open heart surgery: a randomized controlled trial. 2022;21(1):55. **Reason for exclusion: Ineligible participant characteristics** |
| Dadgari A, Rahmani P, Mirrezaie SM. The Effect of Nursing Discharge Planning Program to Prevent Recurrent Falls, Readmission, and Length of Hospital Stay in the Aged Patients: A Randomized Controlled Trial. 2022;38(4):284. **Reason for exclusion: Ineligible participant characteristics** |
| Deer RR, Goodlett SM, Fisher SR, Baillargeon J, Dickinson JM, Raji M, et al. A Randomized Controlled Pilot Trial of Interventions to Improve Functional Recovery After Hospitalization in Older Adults: Feasibility and Adherence. 2018;73(2):193. **Reason for exclusion: Ineligible outcomes** |
| Dhalla IA, O’Brien T, Morra D, Thorpe KE, Wong BM, Mehta R, et al. Effect of a postdischarge virtual ward on readmission or death for high-risk patients: A randomized clinical trial. 2014;312(13):1312.  **Reason for exclusion: Ineligible participant characteristics** |
| Dizon ML, Reinking C. Reducing Readmissions: Nurse-Driven Interventions in the Transition of Care From the Hospital. 2017;14(6):439. **Reason for exclusion: Ineligible participant characteristics** |
| Donzé J, John G, Genné D, Mancinetti M, Gouveia A, Méan M, et al. Effects of a Multimodal Transitional Care Intervention in Patients at High Risk of Readmission: The TARGET-READ Randomized Clinical Trial. 2023;183(7):668. **Reason for exclusion: Ineligible participant characteristics** |
| Emme C, Mortensen EL, Rydahl-Hansen S, Østergaard B, Svarre Jakobsen A, Schou L, et al. The impact of virtual admission on self-efficacy in patients with chronic obstructive pulmonary disease - a randomised clinical trial. 2014;23(21):3137. **Reason for exclusion: Ineligible participant characteristics** |
| Freeman CR, Scott IA, Hemming K, Connelly LB, Kirkpatrick CM, Coombes I, et al. Reducing Medical Admissions and Presentations Into Hospital through Optimising Medicines (REMAIN HOME): a stepped wedge, cluster randomised controlled trial. 2021;214(5):217.  **Reason for exclusion: Ineligible participant characteristics** |
| Goldman LE, Sarkar U, Kessell E, Guzman D, Schneidermann M, Pierluissi E, et al. Support from hospital to home for elders: A Randomized Trial. 2014;161(7):481. **Reason for exclusion: Ineligible participant characteristics** |
| Hall AG, Schumacher JR, Brumback B, Harman JS, Lutz BJ, Hendry P, et al. Health-related quality of life among older patients following an emergency department visit and emergency department-to-home coaching intervention: A randomized controlled trial. 2017;20(4):170. **Reason for exclusion: Ineligible participant characteristics** |
| Hamar B, Rula EY, Wells AR, Coberley C, Pope JE, Varga D. Impact of a scalable care transitions program for readmission avoidance. 2016;22(1):34. **Reason for exclusion: Ineligible participant characteristics** |
| Hamar B, Rula EY, Wells AR, Coberley C, Pope JE, Varga D. Impact of a scalable care transitions program for readmission avoidance. 2016;22(1):34.  **Reason for exclusion: Ineligible participant characteristics** |
| Hamar GB, Coberley C, Pope JE, Cottrill A, Verrall S, Larkin S, et al. Effect of post-hospital discharge telephonic intervention on hospital readmissions in a privately insured population in Australia. 2018;42(3):247. **Reason for exclusion: Ineligible participant characteristics** |
| Hansen LO, Greenwald JL, Budnitz T, Howell E, Halasyamani L, Maynard G, et al. Project BOOST: Effectiveness of a multihospital effort to reduce rehospitalization. 2013;8(8):427. **Reason for exclusion: Ineligible participant characteristics** |
| Hawes EM, Maxwell WD, White SF, Mangun J, Lin F-C. Impact of an outpatient pharmacist intervention on medication discrepancies and health care resource utilization in posthospitalization care transitions. 2014;5(1):18. **Reason for exclusion: Ineligible participant characteristics** |
| Heaton PC, Frede S, Kordahi A, Lowery L, Moorhead B, Kirby J, et al. Improving care transitions through medication therapy management: A community partnership to reduce readmissions in multiple health-systems. 2019;59(3):328. **Reason for exclusion: Ineligible participant characteristics** |
| Heo M, Taaffe K, Ghadshi A, Teague LD, Watts J, Lopes SS, et al. Effectiveness of Transitional Care Program among High-Risk Discharged Patients: A Quasi-Experimental Study on Saving Costs, Post-Discharge Readmissions and Emergency Department Visits. 2023;20(23). **Reason for exclusion: Ineligible participant characteristics** |
| Hohmann C, Neumann-Haefelin T, Klotz JM, Freidank A, Radziwill R. Providing systematic detailed information on medication upon hospital discharge as an important step towards improved transitional care. 2014;39(3):291. **Reason for exclusion: Ineligible participant characteristics** |
| Hoover C, Plamann J, Beckel J. Outcomes of an interdisciplinary transitional care quality improvement project on self-management and health care use in patients with heart failure. 2017;43(1):31. **Reason for exclusion: Ineligible participant characteristics** |
| Hørdam B, Boolsen MW. Patient involvement in own rehabilitation after early discharge. 2017;31(4):866.  **Reason for exclusion: Ineligible participant characteristics** |
| Jacobsohn GC, Jones CMC, Green RK, Cochran AL, Caprio TV, Cushman JT, et al. Effectiveness of a care transitions intervention for older adults discharged home from the emergency department: A randomized controlled trial. 2022;29(1):63.  **Reason for exclusion: Ineligible participant characteristics** |
| Jehloh L, Songwathana P, Kitrungrote L. Transitional Care Based e-Health Program for Older Muslim Thai Adults with Chronic Obstructive Pulmonary Disease After Hospital Discharge: A Feasibility Study. 2024;28(1):115. **Reason for exclusion: Ineligible participant characteristics** |
| Jošt M, Knez L, Mrhar A, Kerec Kos M. Adverse drug events during transitions of care: Randomized clinical trial of medication reconciliation at hospital admission. 2022;134(3):138. **Reason for exclusion: Ineligible participant characteristics** |
| Kapoor A, Landyn V, Wagner J, Burgwinkle P, Huang W, Gore J, et al. Supplying Pharmacist Home Visit and Anticoagulation Professional Consultation during Transition of Care for Patients with Venous Thromboembolism. 2020;16(4):E375. **Reason for exclusion: Ineligible participant characteristics** |
| Kim H-J, Park Y-H. The Effects of Discharge Planning for the Elderly with Pulmonary Disease in the Emergency Room. 2014;7(1):32.  **Reason for exclusion: Language** |
| Kripalani S, Chen G, Ciampa P, Theobald C, Cao A, McBride M, et al. A transition care coordinator model reduces hospital readmissions and costs. 2019;81:61.  **Reason for exclusion: Ineligible participant characteristics** |
| LaBedz SL, Prieto-Centurion V, Mutso A, Basu S, Bracken NE, Calhoun EA, et al. Pragmatic Clinical Trial to Improve Patient Experience Among Adults During Transitions from Hospital to Home: the PArTNER study. 2022;37(16):4111. **Reason for exclusion: Ineligible participant characteristics** |
| Lainscak M, Kadivec S, Kosnik M, Benedik B, Bratkovic M, Jakhel T, et al. Discharge coordinator intervention prevents hospitalizations in patients with COPD: A randomized controlled trial. 2013;14(6):450.e6.  **Reason for exclusion: Ineligible participant characteristics** |
| Li F, Guo J, Suga-Nakagawa A, Takahashi LK, Renaud J. The impact of Kaua’i Care Transition Intervention on hospital readmission rates. 2015;21(10):566. **Reason for exclusion: Ineligible participant characteristics** |
| Liss DT, Ackermann RT, Cooper A, Finch EA, Hurt C, Lancki N, et al. Effects of a Transitional Care Practice for a Vulnerable Population: a Pragmatic, Randomized Comparative Effectiveness Trial. 2019;34(9):1765. **Reason for exclusion: Ineligible participant characteristics** |
| Lou VW, Cheng CY-M, Ng DK-S, Chan FH-W, Mo SS-L, Kung EK-M, et al. A mHealth-Supported Volunteer-Assisted Spiritual Well-Being Intervention for Discharged Older Patients: A Tripartite Collaboration. 2023;66(2):207.  **Reason for exclusion: Ineligible participant characteristics** |
| Low LL, Vasanwala FF, Ng LB, Chen C, Lee KH, Tan SY. Effectiveness of a transitional home care program in reducing acute hospital utilization: a quasi-experimental study. 2015;15:100. **Reason for exclusion: Ineligible participant characteristics** |
| Markle-Reid M, Valaitis R, Bartholomew A, Fisher K, Fleck R, Ploeg J, et al. An integrated hospital-to-home transitional care intervention for older adults with stroke and multimorbidity: A feasibility study. 2020;10:N.PAG. **Reason for exclusion: Ineligible participant characteristics** |
| Menchine M, Oberfoell S, Schriger D, Walker C, Riddell J, Arora S. Improving telephone follow-up for patients discharged from the emergency department: Results of a randomized controlled trial. 2013;20(5):462.  **Reason for exclusion: Ineligible participant characteristics** |
| Mi R, Hollander MM, Jones CMC, Dugoff EH, Caprio TV, Cushman JT, et al. A randomized controlled trial testing the effectiveness of a paramedic-delivered care transitions intervention to reduce emergency department revisits. 2018;18(1.  **Reason for exclusion: Ineligible study design** |
| Neu R, Leonard MA, Dehoorne ML, Scalia SJ, Kale-Pradhan PB, Giuliano CA. Impact of Pharmacist Involvement in Heart Failure Transition of Care. 2020;54(3):246.  **Reason for exclusion: Ineligible participant characteristics** |
| Nguyen AT, Wisniewski J, Leang DW, Keller MS, Rosen S, Shane R, et al. Effect of the population health inpatient Medicare Advantage pharmacist intervention on hospital readmissions: A quasi-experimental controlled study. 2023;29(3):275. **Reason for exclusion: Ineligible participant characteristics** |
| Nguyen PAA, Enwere E, Gautreaux S, Lin H, Tverdek F, Lu M, et al. Impact of a pharmacy-driven transitions-of-care program on postdischarge healthcare utilization at a national comprehensive cancer center. 2018;75(18):1393.  **Reason for exclusion: Ineligible participant characteristics** |
| Odeh M, Scullin C, Fleming G, Scott MG, Horne R, McElnay JC. Ensuring continuity of patient care across the healthcare interface: Telephone follow-up post-hospitalization. 2019;85(3):625. **Reason for exclusion: Ineligible participant characteristics** |
| Ohuabunwa U, Jordan Q, Shah S, Fost M, Flacker J. Implementation of a care transitions model for low-income older adults: A high-risk, vulnerable population. 2013;61(6):992. **Reason for exclusion: Ineligible participant characteristics** |
| Pannill FC. In older hospitalized patients, adding transitional care to in-hospital geriatric assessment did not improve ADL. 2016;164(12):1. **Reason for exclusion: Ineligible study design** |
| Reid M, Valaitis R, Bartholomew A, Fisher K, Fleck R, Ploeg J, et al. Implementation and evaluation of an integrated hospital-to-home transitional care intervention for older adults with stroke and multimorbidity: a feasibility study. 2019;19:2. **Reason for exclusion: Ineligible study design** |
| Ritchie CS, Houston TK, Richman JS, Sobko HJ, Berner ES, Taylor BB, et al. The E-Coach technology-assisted care transition system: a pragmatic randomized trial. 2016;6(3):437. **Reason for exclusion: Ineligible participant characteristics** |
| Santana RF, Pereira SK, do Carmo TG, Freire VEC de S, Soares T da S, do Amaral DM, et al. Effectiveness of a telephone follow-up nursing intervention in postsurgical patients. 2018;24(4):e12648. **Reason for exclusion: Ineligible participant characteristics** |
| Shah MN, Jacobsohn GC, Jones CMC, Green RK, Caprio TV, Cochran AL, et al. Care transitions intervention reduces ED revisits in cognitively impaired patients. 2022;8(1).  **Reason for exclusion: Ineligible participant characteristics** |
| Taylor SP, Murphy S, Rios A, McWilliams A, McCurdy L, Chou S-H, et al. Effect of a Multicomponent Sepsis Transition and Recovery Program on Mortality and Readmissions after Sepsis: The Improving Morbidity during Post-Acute Care Transitions for Sepsis Randomized Clinical Trial∗. 2022;50(3):479. **Reason for exclusion: Ineligible participant characteristics** |
| Ko, Y., Hwang, J. M., & Baek, S. H. (2023). Discharge transitional care programme for older adults after hip fracture surgery: a quasi-experimental study.*Journal of research in nursing : JRN*, *28*(8), 582–593. **Reason for exclusion: Ineligible study design** |
| Tuttle KR, Alicic RZ, Short RA, Neumiller JJ, Gates BJ, Daratha KB, et al. Medication therapy management after hospitalization in CKD: A randomized clinical trial. 2018;13(2):241.  **Reason for exclusion: Ineligible participant characteristics** |
| Van Hollebeke M, Talavera-Pons S, Mulliez A, Sautou V, Bommelaer G, Abergel A, et al. Impact of medication reconciliation at discharge on continuity of patient care in France. 2016;38(5):1156. **Reason for exclusion: Ineligible outcomes** |
| Vergara FH, Davis JE, Budhathoki C, Sullivan NJ, Sheridan DJ. Face-to-Face Meetings with Neurosurgical Patients Before Hospital Discharge: Impact on Telephone Outreach, Emergency Department Visits, and Hospital Readmissions. 2020;23(2):182**Reason for exclusion: Ineligible participant characteristics** |
| Wingard RL, McDougall K, Axley B, Howard A, Okeefe C, Armistead N, et al. Right TraC™ Post-Hospitalization Care Transitions Program to Reduce Readmissions for Hemodialysis Patients. 2017;45(6):539.  **Reason for exclusion: Ineligible participant characteristics** |
| Xu Y, Li S, Zhao P, Zhao J. Using the knowledge-to-action framework with joint arthroplasty patients to improve the quality of care transition: A quasi-experimental study. 2020;15(1). **Reason for exclusion: Ineligible participant characteristics** |
| Yiadom MYAB, Domenico HJ, Byrne DW, Hasselblad M, Kripalani S, Choma N, et al. Impact of a follow-up telephone call program on 30-day readmissions (FUTR-30): A pragmatic randomized controlled real-world effectiveness trial. 2020;58(9):792. **Reason for exclusion: Ineligible participant characteristics** |
| Zatzick D, Russo J, Thomas P, Darnell D, Teter H, Ingraham L, et al. Patient-Centered Care Transitions After Injury Hospitalization: A Comparative Effectiveness Trial. 2018;81(2):157. **Reason for exclusion: Ineligible participant characteristics** |
| van der Vlegel-Brouwer W, Breman P, Thomas L, Macphail L. A quasi experimental before and after study of a transitional care programme for older adults in the area of the IJsselland Hospital. 2018;18:2. **Reason for exclusion: Ineligible study design** |
| Torisson G, Stavenow L, Minthon L, Londos E. Effects of a comprehensive geriatric assessment on frail elderly patients discharged from an acute medical unit: a randomized controlled trial. 2013;13:228.  **Reason for exclusion: Ineligible study design** |
| Nielsen LM, Maribo T, Kirkegaard H, Petersen KS, Lisby M, Oestergaard LG. Effectiveness of the “Elderly Activity Performance Intervention” on elderly patients’ discharge from a short-stay unit at the emergency department: a quasi-experimental trial. *Clinical Interventions in Aging*. 2018;13:737–747. **Reason for exclusion: Ineligible study design** |
| Heeren P, Devriendt E, Fieuws S, Wellens NIH, Deschodt M, Flamaing J, et al. Unplanned readmission prevention by a geriatric emergency network for transitional care (URGENT): a prospective before-after study. BMC Geriatr. 2019;19:215. **Reason for exclusion: Ineligible study design** |
| Low, L. L., Vasanwala, F. F., Ng, L. B., Chen, C., Lee, K. H., & Tan, S. Y. (2015). Effectiveness of a transitional home care program in reducing acute hospital utilization: a quasi-experimental study. *BMC health services research*, *15*, 100. **Reason for exclusion: Ineligible study design** |
| Rasmussen LJH, Petersen J, Johnsen SP, Skjødt U, Beyer N, Kristensen MT, et al. Hospital-initiated rehabilitation following short acute admissions in older patients: a randomized controlled trial. *BMC Geriatr.* 2022;22:319. **Reason for exclusion: Ineligible study design** |
| Ong C, Asinas-Tan M, Quek LS, de Dios M, Cai XT, Lim BL. Effectiveness of a post-emergency department discharge multidisciplinary bundle in reducing acute hospital admissions for the elderly. *European Journal of Emergency Medicine*. 2017;00:000–000. **Reason for exclusion: Ineligible study design** |
| Chang PC, Li YC, Chen HJ, Huang HC, Chen W, Kuo YC, et al. Effectiveness of using telephone follow-up intervention on the unplanned readmission of older adult patients within 14 days of being discharged from hospital. *International Journal of Gerontology*. 2023;17(4):226–231. **Reason for exclusion: Ineligible study design** |
| Chong E, Zhu B, Tan H, De Castro Molina J, Goh EF, Baldevarona-Llego J, et al. Emergency Department Interventions for Frailty (EDIFY): Front-Door Geriatric Care Can Reduce Acute Admissions. 2021;22:923–928. **Reason for exclusion: Ineligible study design** |
| Conneely M, O’Shea E, O’Neill D, Blake C, Irving K, McHugh S, et al. Evaluation of a transitional care intervention for older adults (the ACTION study): a quasi-experimental effectiveness–implementation study. *BMC Geriatr.* 2023;23:240. **Reason for exclusion: Ineligible study design** |
| Han CY, Liu MF, Tsay SF, Lee CY. The effect of transitional care on the readmission rate of older medical patients: a quasi-experimental study. *International Journal of Nursing Practice*. 2023;29(3):e13125. **Reason for exclusion: Ineligible study design** |
| Finlayson, K., Chang, A. M., Courtney, M. D., Edwards, H. E., Parker, A. W., Hamilton, K., Pham, T. D. X., & O’Brien, J. (2018). Transitional care interventions reduce unplanned hospital readmissions in high-risk older adults. *BMC Health Services Research, 18*(956).  **Reason for exclusion: Ineligible study design** |
| González-Ortega, I., de Pedro-Gómez, J. E., Rodríguez-Gonzalo, A., & Morales-Asencio, J. M. (2017). Effectiveness of discharge planning in primary health care for post-surgical patients. *Journal of Clinical Nursing, 26*(23–24), 4750–4763.  **Reason for exclusion: Ineligible study design** |
| Padula, W. V., Laveist, T. A., & Velopulos, C. (2019). A cost-effectiveness analysis of transitional care services for Medicare beneficiaries. *BMC Health Services Research, 19*, 921.  **Reason for exclusion: Ineligible study design** |
| Haag-Heitman, B., & Kramer, D. (2016). Creating infrastructure and evidence for transitional care. *Nursing Administration Quarterly, 40*(2), 138–147. **Reason for exclusion: Ineligible study design** |
| Thygesen, L. C., Paulsen, M. S., Möbius, N., Christensen, M. B., & Søndergaard, J. (2015). Continuity of care for recently discharged patients—a population-based study. *British Journal of General Practice, 65*(640), e264–e271. **Reason for exclusion: Ineligible study design** |

Appendix 3: Intervention component and transition stage

| **Intervention components of the studies grouped according to TCM (Naylor et al. 2009) and COMET taxonomy (Dodd et al. 2023)** | Alizadeh-Khoei et al. 2023 | Altfeld et al. 2013 | Arendts et al. 2018 | Berglund et al. 2015 | K. Biese et al. 2014 | K. J. Biese et al. 2018 | Blondal et al. 2023 | Buurman et al. 2016 | Clemson et al. 2016 | Deer et al. 2019 | Deutz et al. 2016 | Finlayson et al. 2018 | Grahn et al. 2019 | Gurwitz et al. 2014 | Jepma et al. 2021 | Kempen et al. 2021 | Lee et al. 2023 | Lembeck et al 2019 | Lindegaard-Pedersen et al. 2017 | Lockwood et al. 2019 | Ong et al. 2016 | Ozaki et al. 2023 | Schapira et al. 2022 | Van Spall et al. 2019 | Xueyu et al. 2017 | *Components total (N)* |
| --- | --- | --- | --- | --- | --- | --- | --- | --- | --- | --- | --- | --- | --- | --- | --- | --- | --- | --- | --- | --- | --- | --- | --- | --- | --- | --- |
| PRE-HOSPITALISATION |  |  |  |  |  |  |  |  |  |  |  |  |  |  |  |  |  |  |  |  |  |  |  |  |  |  |
| Preadmission early care referral |  |  |  |  |  |  |  |  |  |  |  |  |  |  |  |  |  |  |  |  |  | x |  |  |  | 1 |
| DURING HOSPITALISATION |  |  |  |  |  |  |  |  |  |  |  |  |  |  |  |  |  |  |  |  |  |  |  |  |  |  |
| Medication reconciliation / review | x | x | x | x | x | x | x | x | x |  |  |  | x | x | x | x |  | x | x |  | x | x | x | x |  | 19 |
| Care planning | x | x | x | x | x | x |  |  |  |  |  |  | x | x | x | x |  | x |  |  |  | x | x | x |  | 14 |
| Assessment:   - Geriatric - Nutritional - General /not specified - Exercise | x |  | x |  |  |  | x | x |  | x | x | x | x |  | x |  | x |  |  |  | x |  |  |  |  | 7  2  1  1 |
| Screening for risk-factors | x | x | x | x | x | x | x | x |  |  |  | x | x |  | x | x | x | x | x |  | x | x | x | x | x | 20 |
| Education on self-care and symptom management | x |  |  |  |  |  | x |  |  |  |  | x |  |  |  |  | x |  |  |  | x |  |  |  |  | 5 |
| Discharge coordination | x | x | x | x | x | x | x | x | x | x | x | x | x | x | x | x | x | x | x | x | x | x | x | x | x | 25 |
| POST DISCHARGE |  |  |  |  |  |  |  |  |  |  |  |  |  |  |  |  |  |  |  |  |  |  |  |  |  |  |
| Home safety assessment | x | x |  |  |  |  |  |  |  |  |  |  |  |  |  |  |  |  |  |  |  |  |  |  |  | 2 |
| ADL training/ exercise prescription / exercise plans |  |  |  |  |  |  |  | x | x | x |  | x |  |  |  |  | x | x | x | x |  |  |  |  | x | 9 |
| Nutritional supplements / meal delivery |  |  |  |  |  |  | x |  |  | x | x |  |  |  |  | x |  |  |  |  |  |  |  |  |  | 4 |
| Home visits | x |  | x | x |  |  | x |  | x | x | x | x |  |  | x |  |  |  | x | x | x |  |  | x |  | 13 |
| Telephone follow-ups | x | x | x | x | x | x | x |  | x | x | x | x |  |  |  | x | x |  | x |  | x |  | x | x | x | 19 |
| Medication management | x | x | x | x | x | x |  | x |  |  |  |  | x | x | x | x |  | x |  |  |  |  |  |  |  | 12 |
| Education on treatment and equipment | x | x |  |  |  | x |  |  | X |  |  | x | x |  |  | x | x |  |  | x |  |  | x |  |  | 10 |
| Lifestyle interventions | x |  |  |  |  |  |  |  |  |  |  |  |  |  |  |  |  |  |  |  |  |  |  |  |  | 1 |
| Family / caregiver involvement | X |  |  |  |  |  | x | x | x |  |  | x |  |  |  |  |  |  |  | x |  |  | x | x |  | 8 |
| Telemonitoring / alert system |  |  |  |  |  |  |  |  |  |  |  |  |  | x |  |  |  |  |  |  | X | x |  |  |  | 3 |
| Education on self-management |  |  |  |  |  |  | x |  |  |  |  |  |  |  |  |  | x |  |  |  |  |  | x |  |  | 3 |
| **Total interventions/study (N)** | 13 | 8 | 8 | 7 | 6 | 7 | 10 | 7 | 7 | 6 | 7 | 9 | 7 | 5 | 7 | 8 | 8 | 6 | 6 | 5 | 9 | 5 | 8 | 7 | 4 |  |

Appendix 4: Characteristics of the intervention delivery: duration, frequency, and delivery mode

| **Author** | **Before/at discharge** | **1 week** | **2 weeks** | **3 weeks** | **1 month** | **5 weeks** | **6 weeks** | **7 weeks** | **2 months** | **9 weeks** | **10 weeks** | **11 weeks** | **3 months** | **4 months** | **5 months** | **6 months** | **9 months** | **1 year** |
| --- | --- | --- | --- | --- | --- | --- | --- | --- | --- | --- | --- | --- | --- | --- | --- | --- | --- | --- |
| (Alizadeh-Khoei et al. 2023) | 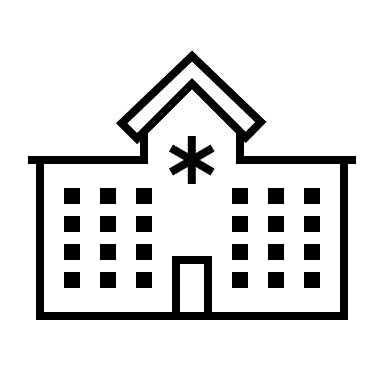 | 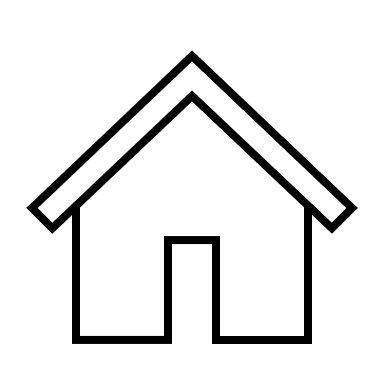 | 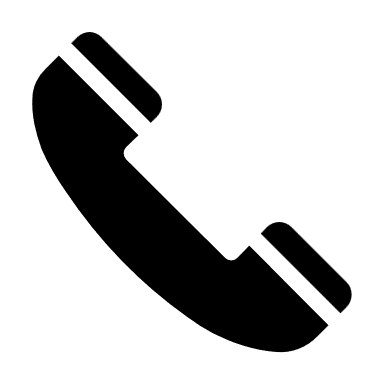 | 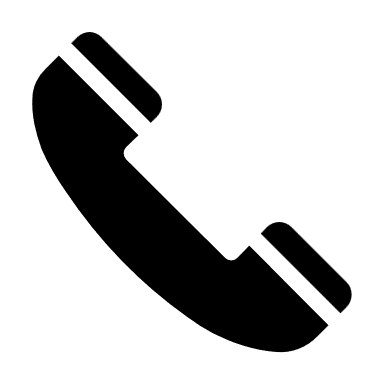 | 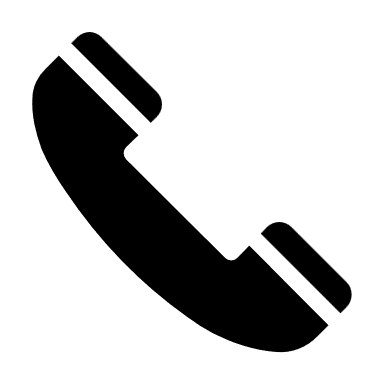 | 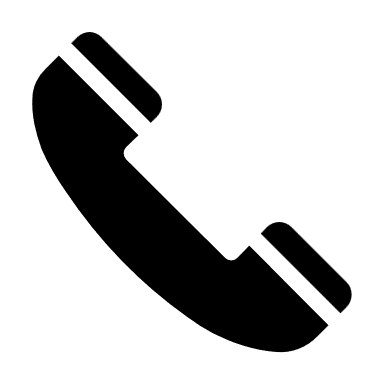 | | | |  |  |  |  |  |  |  |  |  |
| (Altfeld et al., 2013) |  | 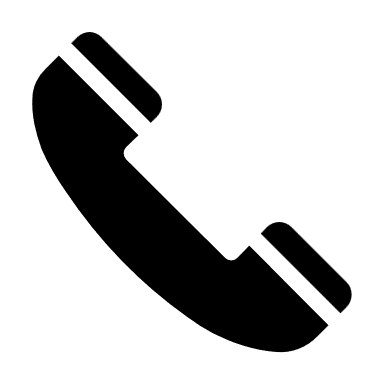 |  |  |  |  |  |  |  |  |  |  |  |  |  |  |  |  |
| (Arendts et al. 2018) |  | 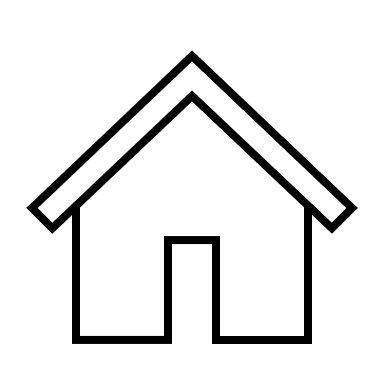 |  | 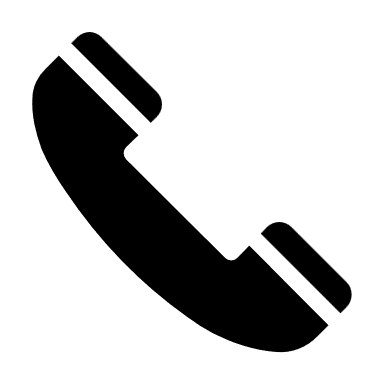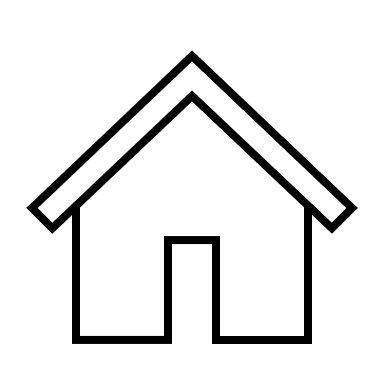^2^ | | | | | | | | | | | | |  |  |
| (Berglund et al. 2015) | 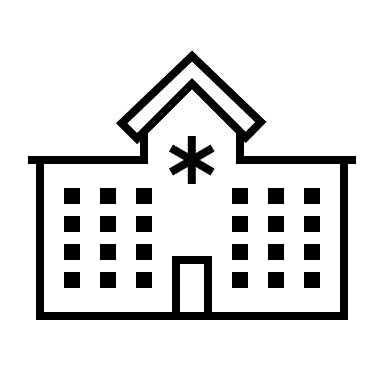 | 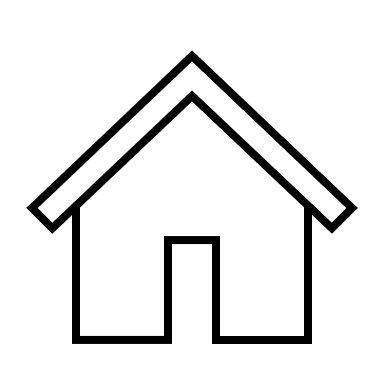 |  |  | 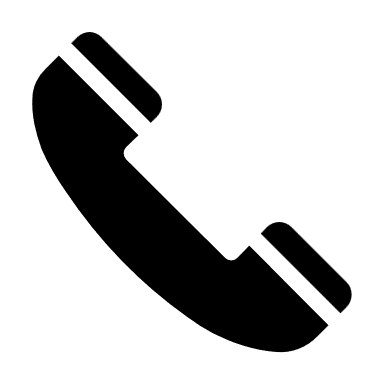^3^ | 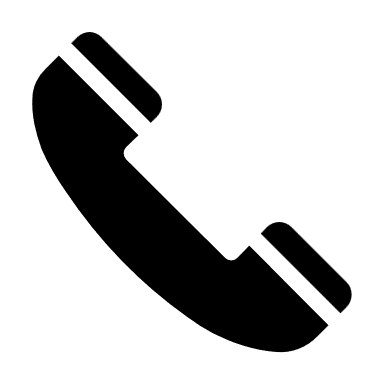 | | | | 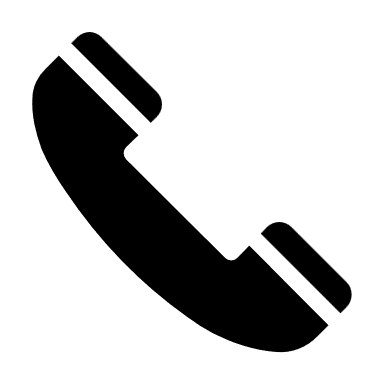 | | | | 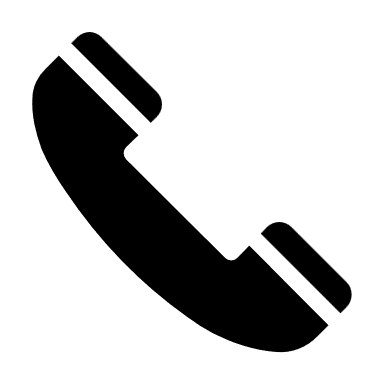 | 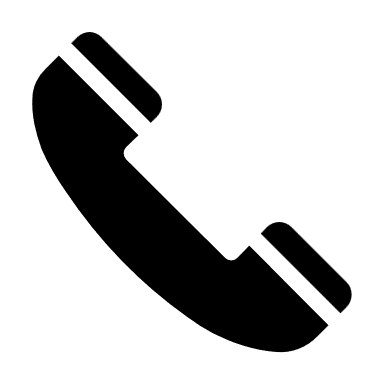 | 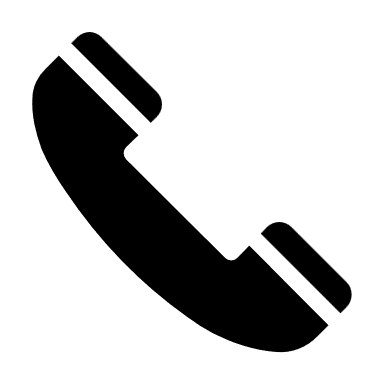 | 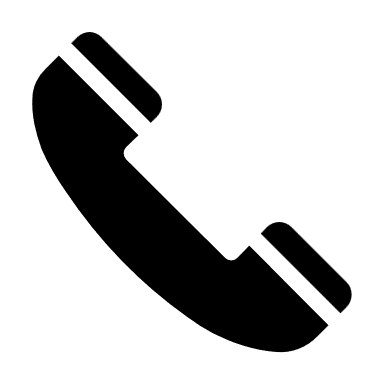 | 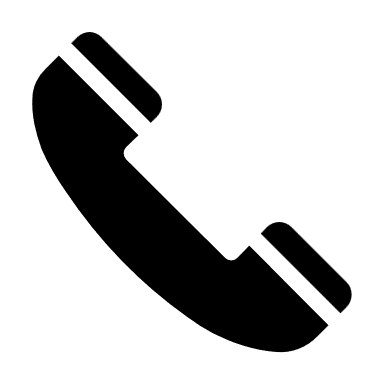 |
| (Biese et al. 2014) |  | 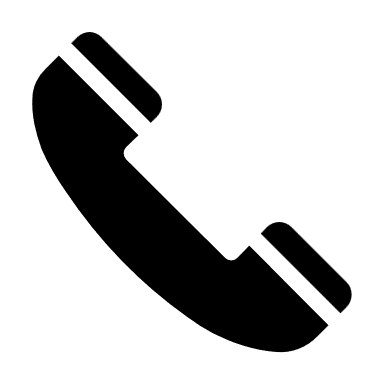 |  |  |  |  |  |  |  |  |  |  |  |  |  |  |  |  |
| (Biese et al. 2018) |  | 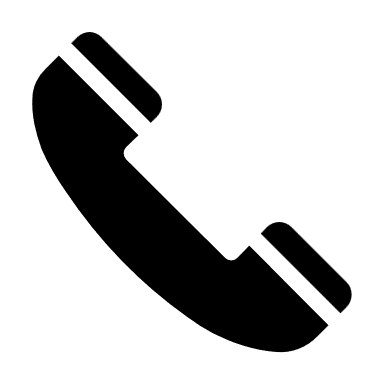 |  |  |  |  |  |  |  |  |  |  |  |  |  |  |  |  |
| (Blondal et al. 2023) |  | 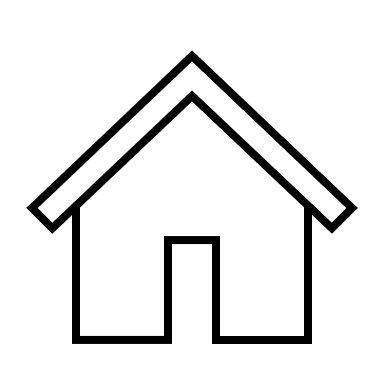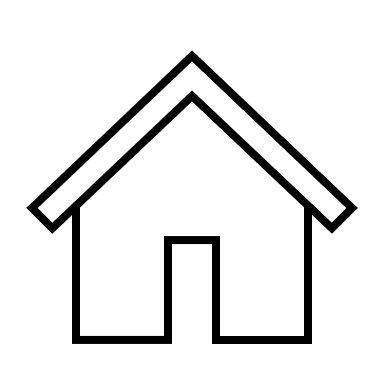 | 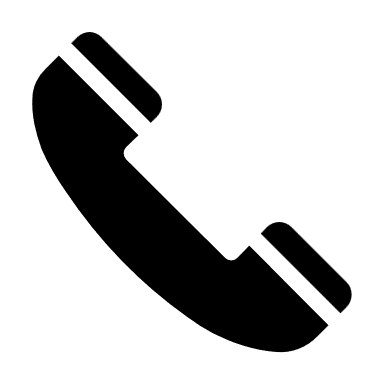 | 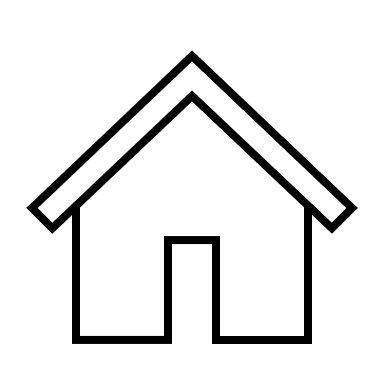 |  | 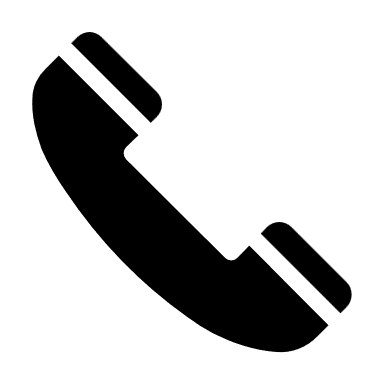 | 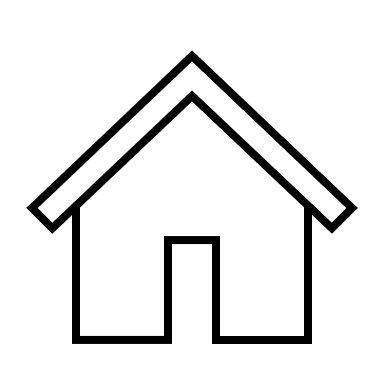 |  |  | 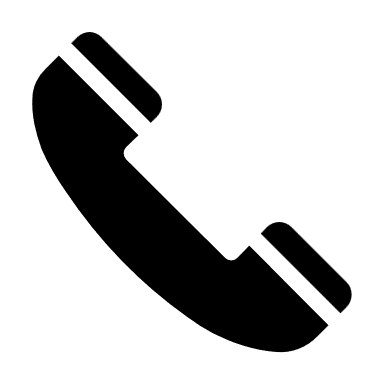 |  |  | 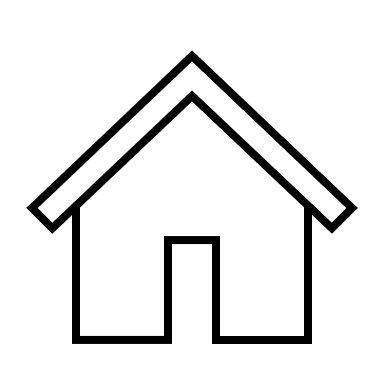 |  |  |  |  |  |
| (Buurman et al. 2016) | 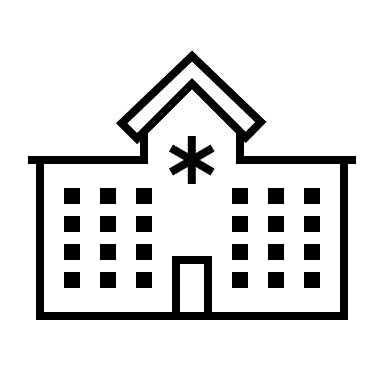 | 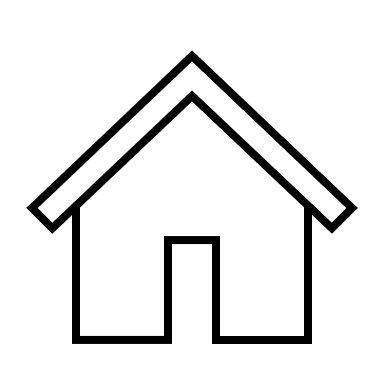 | 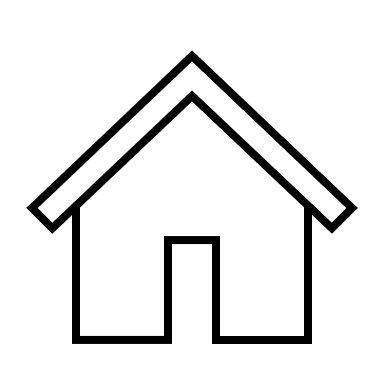 |  |  |  | 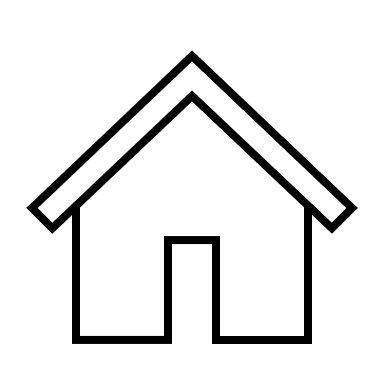 |  |  |  |  |  |  | 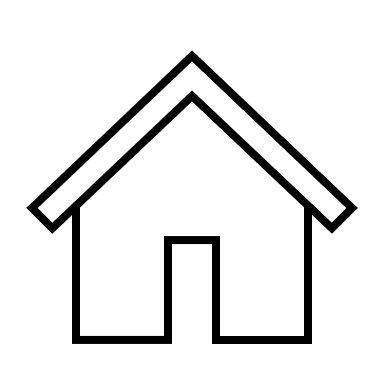 |  | 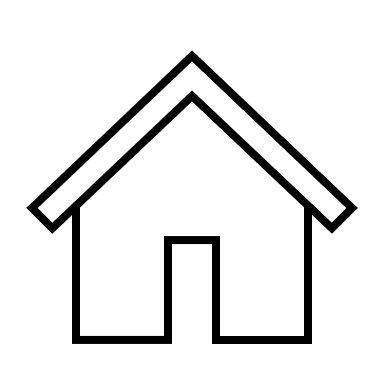 |  |  |
| (Clemson et al. 2016) | 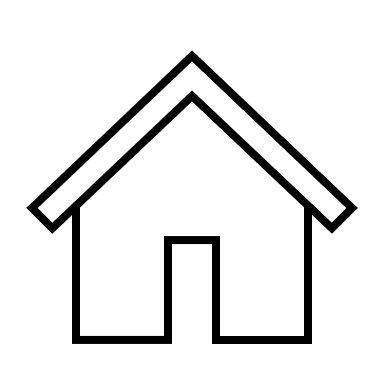 | 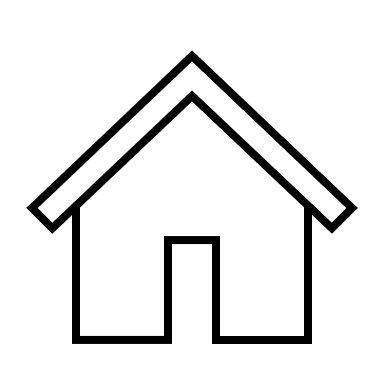 | 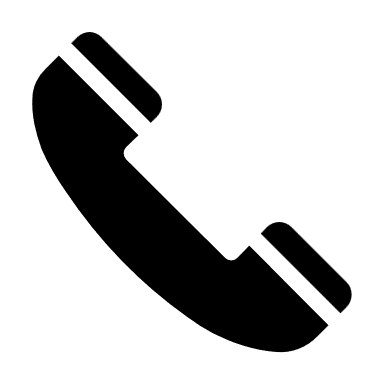 |  | 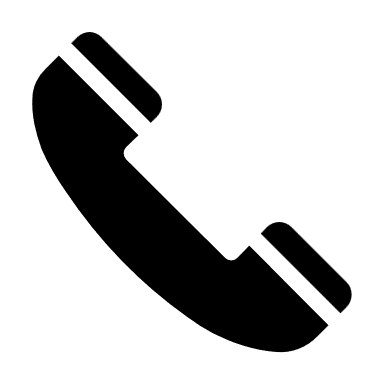 |  |  |  |  |  |  |  |  |  |  |  |  |  |
| (Deer et al. 2019) |  |  | 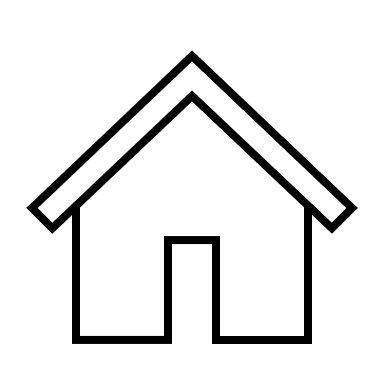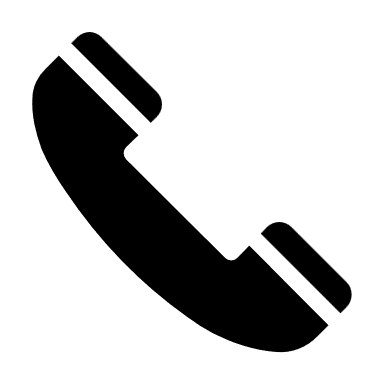^2^ | | |  |  |  |  |  |  |  |  |  |  |  |  |  |
| (Deutz et al. 2016) |  | 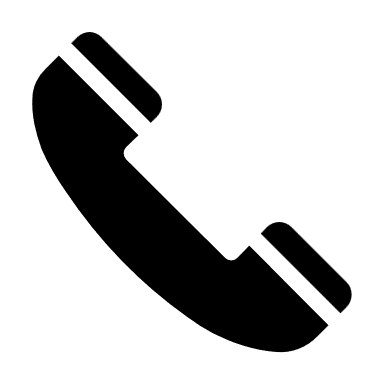/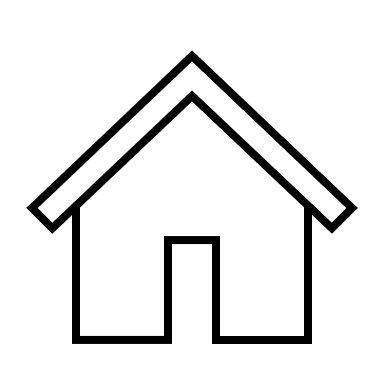 | 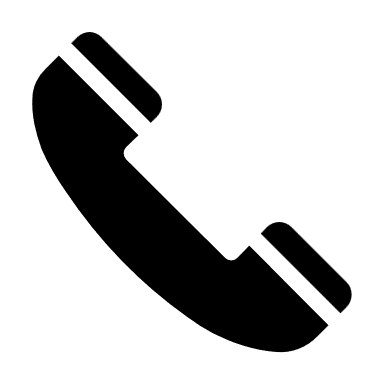/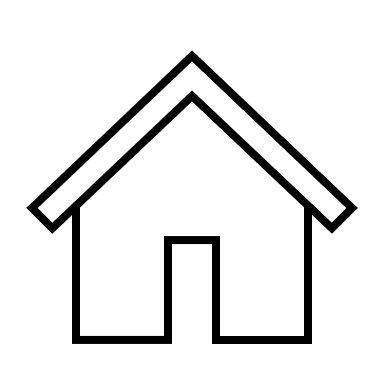 | 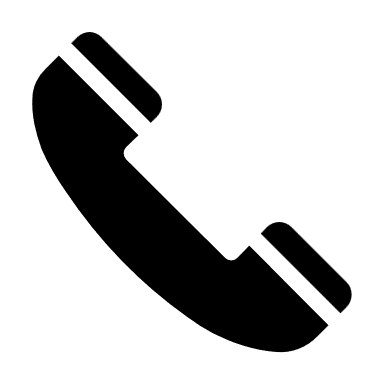/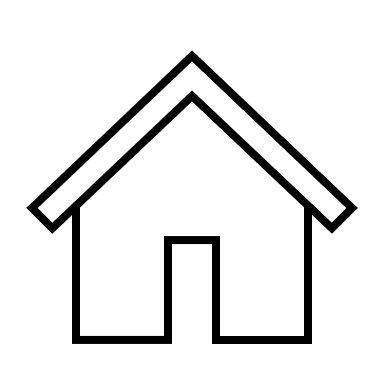 | 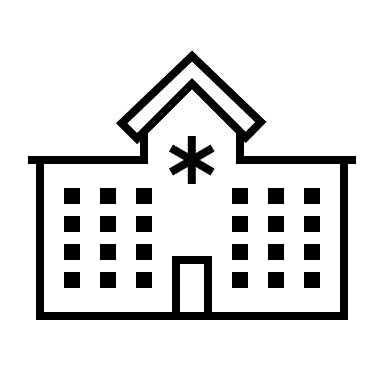 | 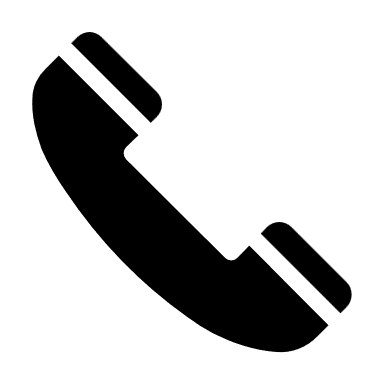/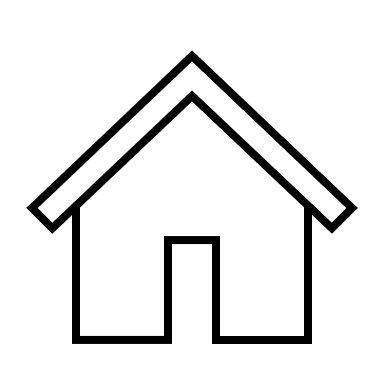 | 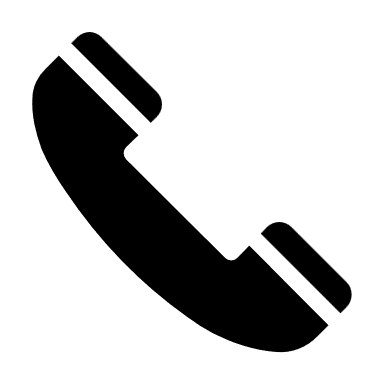/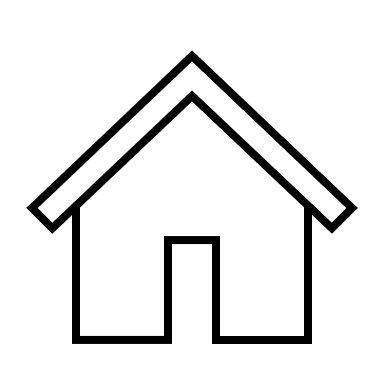 | 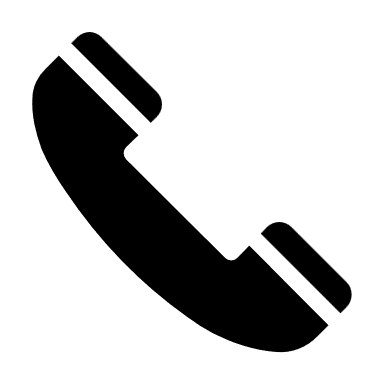/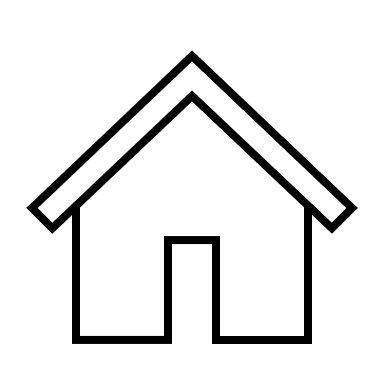 | 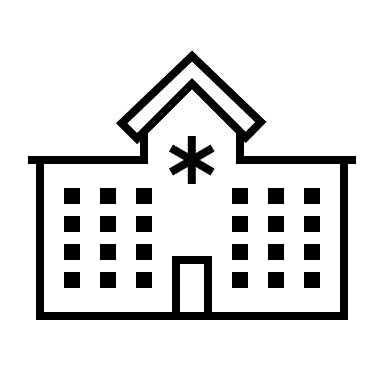 | 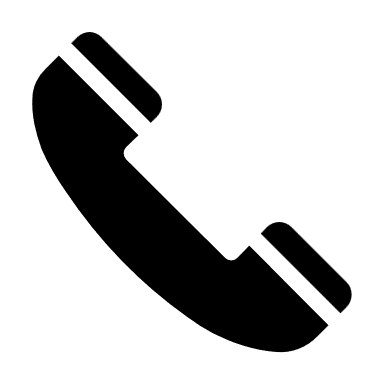/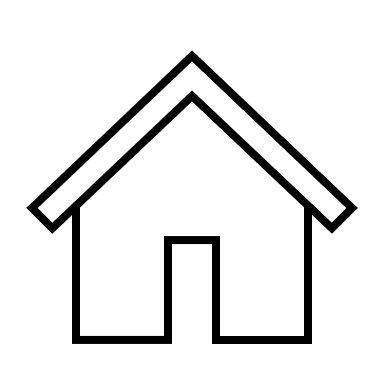 | 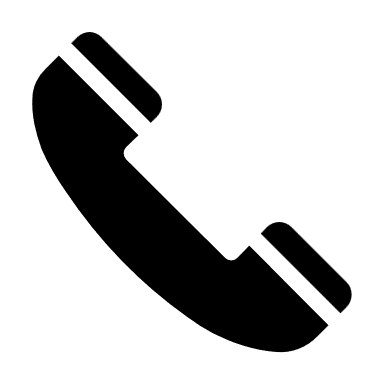/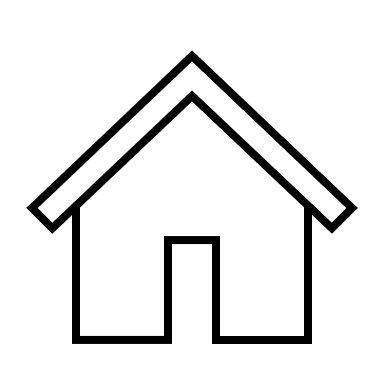 | 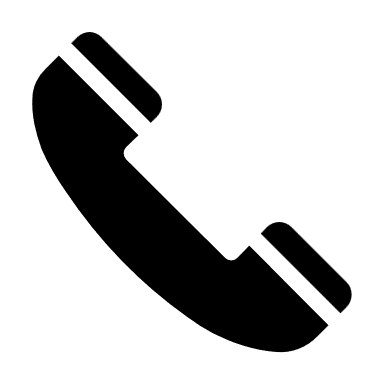/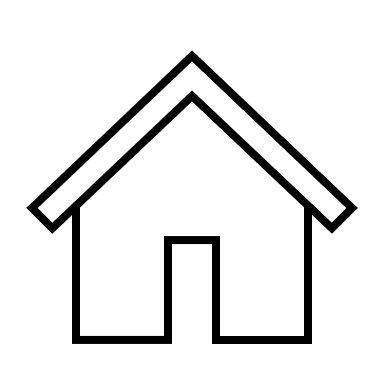 | 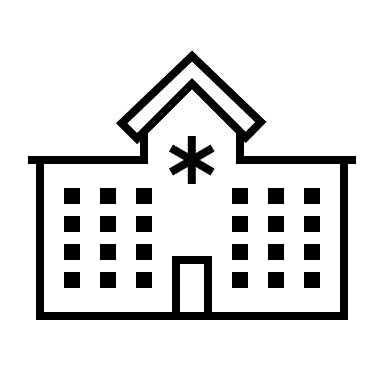 |  |  |  |  |  |
| (Finlayson et al. 2018) | 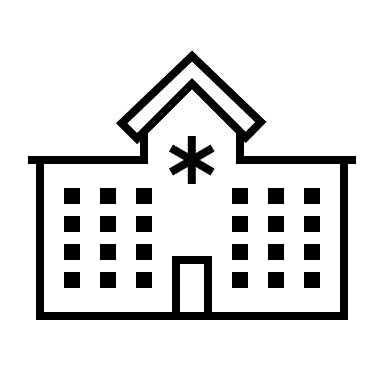 | 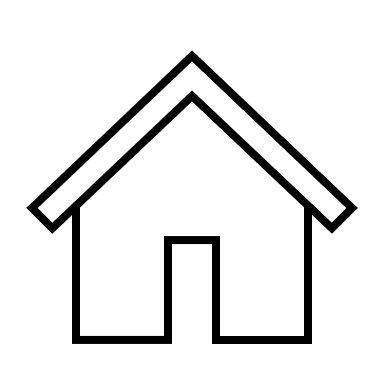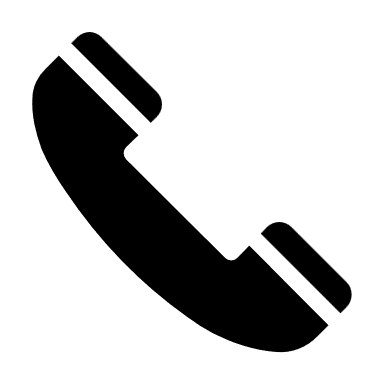 | 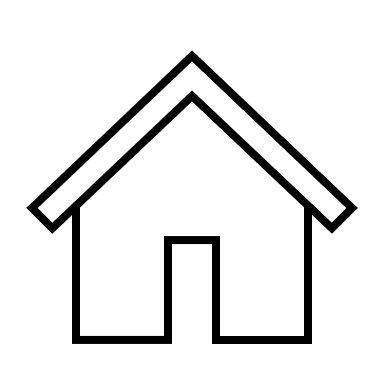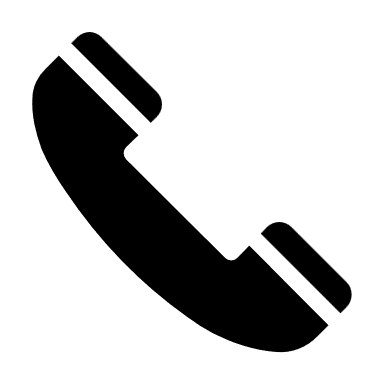 | 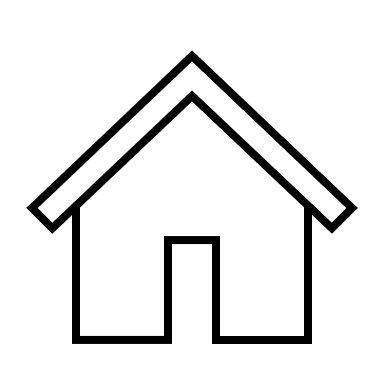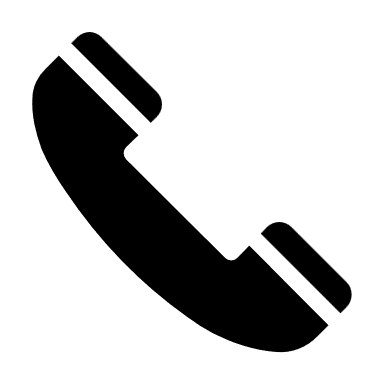 | 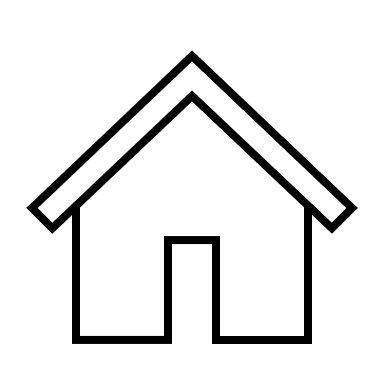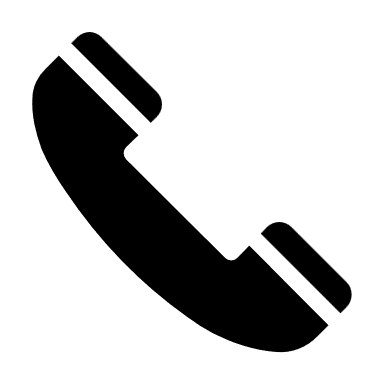 | 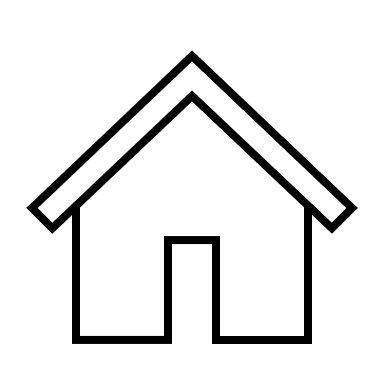 | 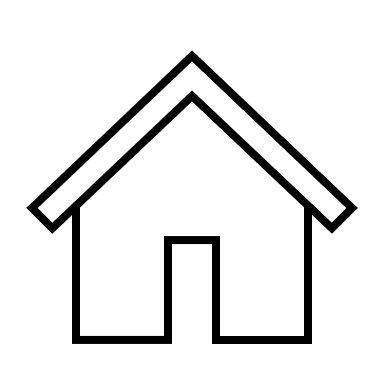 |  | 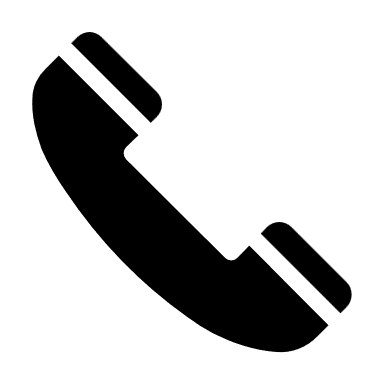 |  |  |  | 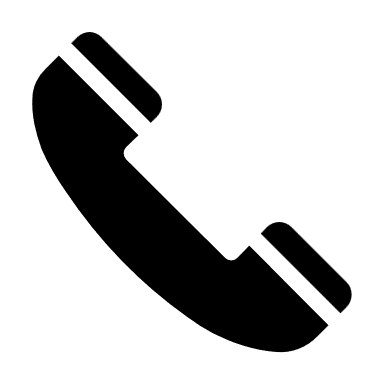 | 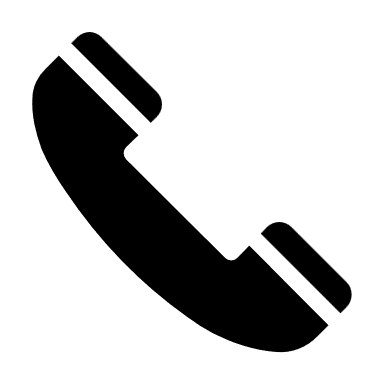 | 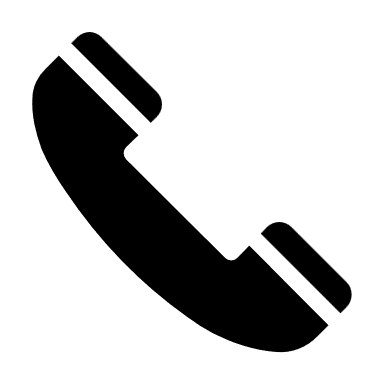 | 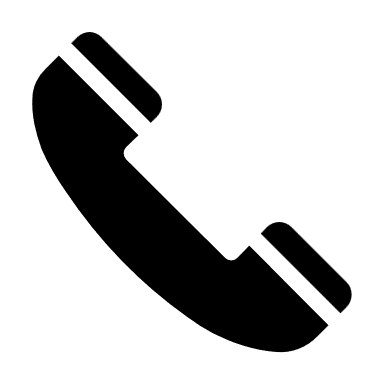 |  |  |
| (Grahn et al. 2019) | 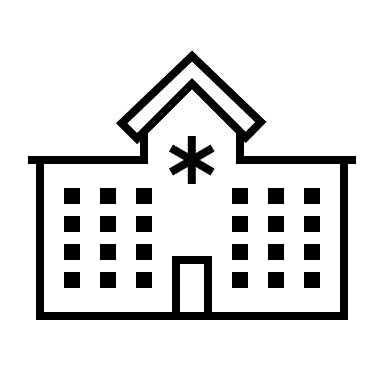 | 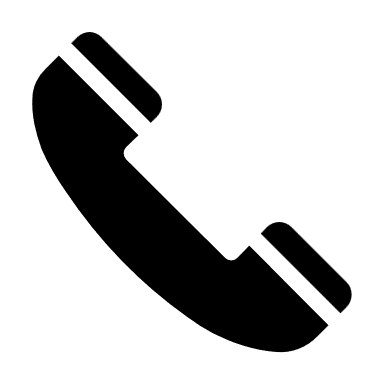 |  |  |  |  |  |  |  |  |  |  |  |  |  |  |  |  |
| (Gurwitz et al. 2014) |  | 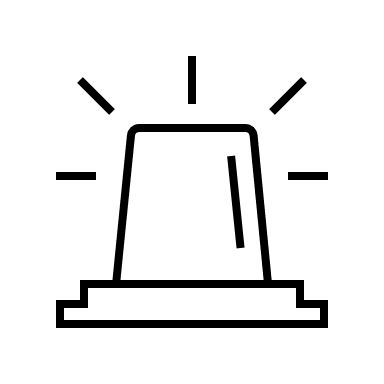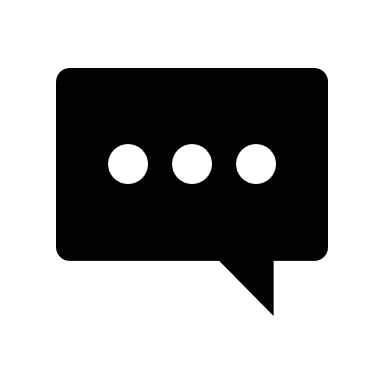 |  |  |  |  |  |  |  |  |  |  |  |  |  |  |  |  |
| (Jepma et al. 2021) | 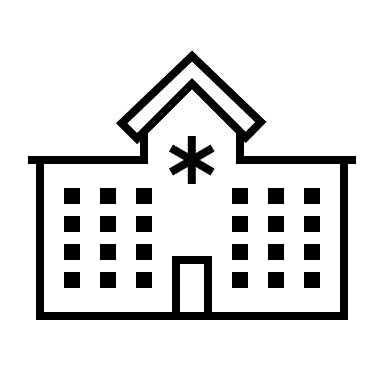 | 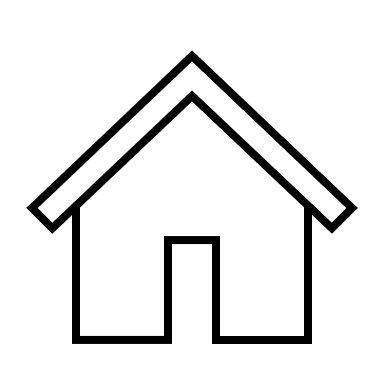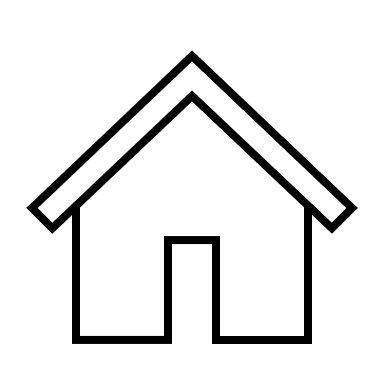 |  | 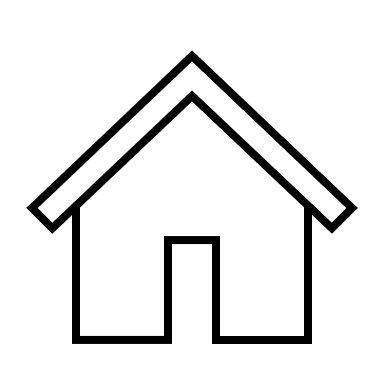 |  |  | 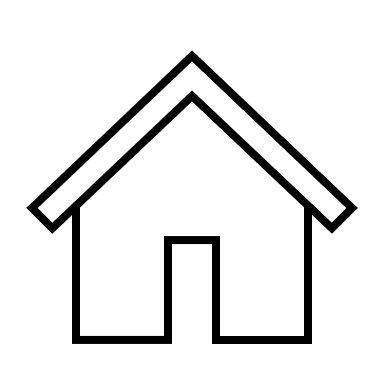 |  |  |  |  |  |  |  |  |  |  |  |
| (Kempen et al. 2021) | 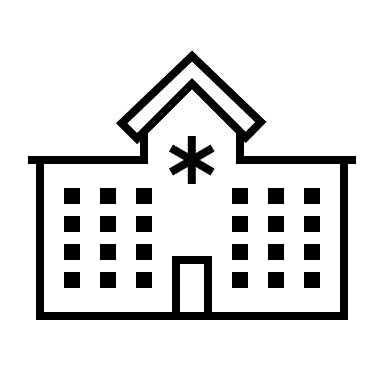 | 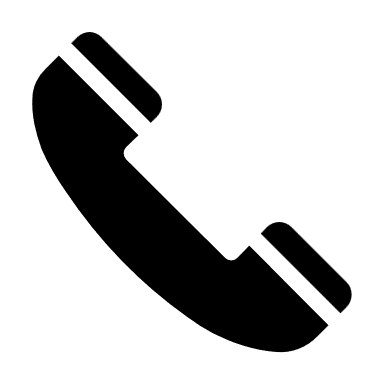^1^ |  |  | 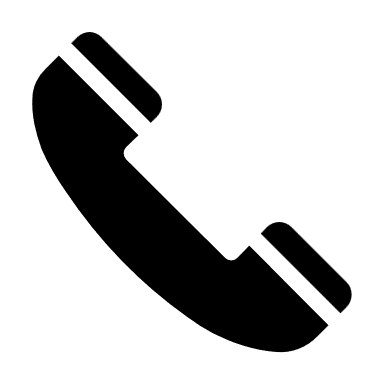^1^ |  |  |  | 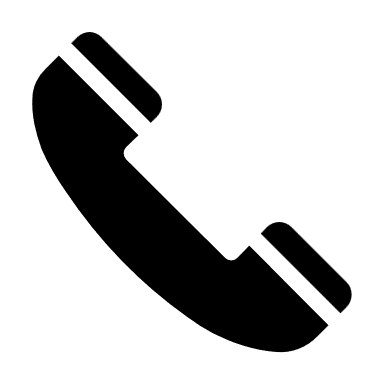^1^ |  |  |  |  |  |  |  |  |  |
| (Lee et al. 2023) | 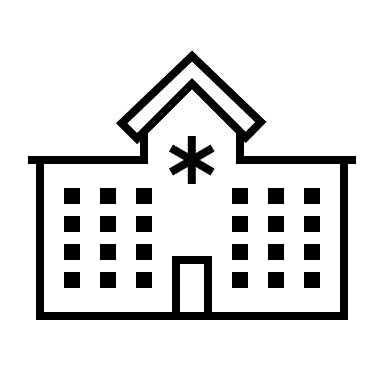 |  | 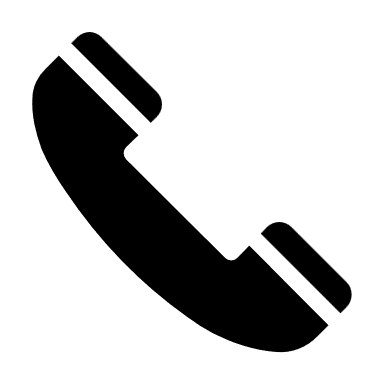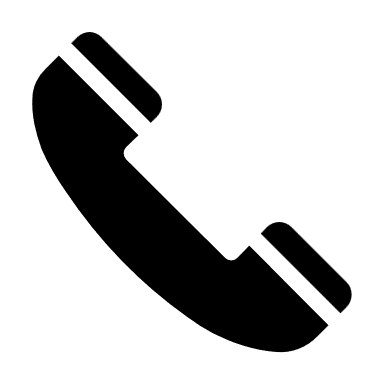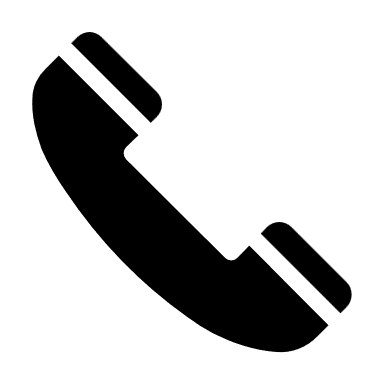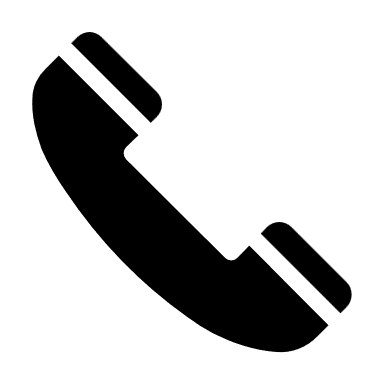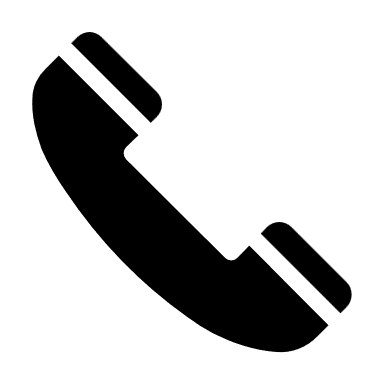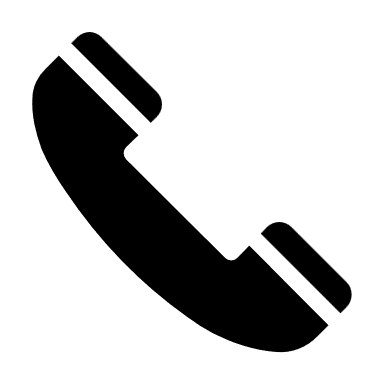 | | | | | | | | | | |  |  |  |  |  |
| (Lembeck et al. 2019) | 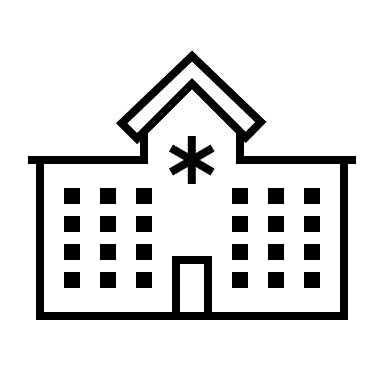 |  |  |  |  |  |  |  |  |  |  |  |  |  |  |  |  |  |
| (Lindegaard-Pedersen et al. 2017) |  | / | / |  | / |  |  |  |  |  |  |  |  |  |  |  |  |  |
| (Lockwood et al. 2019) |  |  |  |  |  |  |  |  |  |  |  |  |  |  |  |  |  |  |
| (Ong et al. 2016) |  |  |  |  |  |  |  |  |  |  |  |  |  |  |  |  |  |  |
| (Ozaki et al. 2023) |  |  |  |  |  |  |  |  |  |  |  |  |  |  |  |  |  |  |
| (Schapira et al. 2022) |  |  |  |  |  |  |  |  |  |  |  |  |  |  |  |  |  |  |
| (Van Spall et al. 2019) |  |  |  |  |  |  |  |  |  |  |  |  |  |  |  |  |  |  |
| (Xueyu et al. 2017) |  |  |  |  |  |  | | | |  | | | |  |  |  |  |  |

Note: = acute care setting visit, = home visit, =telephone, = alert, =messaging, “/” = OR the patients’ needs Legend: ^1^ = only in the intervention 2 group, ^2^ = frequency not specified
